# Metabolic reprogramming during *Candida albicans* planktonic-biofilm transition is modulated by the transcription factors Zcf15 and Zcf26

**DOI:** 10.1371/journal.pbio.3002693

**Published:** 2024-06-21

**Authors:** Laxmi Shanker Rai, Murielle Chauvel, Hiram Sanchez, Lasse van Wijlick, Corinne Maufrais, Thomas Cokelaer, Natacha Sertour, Mélanie Legrand, Kaustuv Sanyal, David R. Andes, Sophie Bachellier-Bassi, Christophe d’Enfert

**Affiliations:** 1 Institut Pasteur, Université Paris Cité, INRAE USC2019, Unité Biologie et Pathogénicité Fongiques, Paris, France; 2 Department of Life Sciences, GITAM University, Bengaluru, Karnataka 561203, India; 3 Department of Medicine, University of Wisconsin, Madison, Wisconsin, United States of America; 4 Institut Pasteur, Université Paris Cité, Hub de Bioinformatique et Biostatistique, Paris, France; 5 Molecular Mycology Laboratory, Molecular Biology and Genetics Unit, Jawaharlal Nehru Centre for Advanced Scientific Research, Jakkur, Bangalore, India; 6 Department of Biological Sciences, Bose Institute, Unified Academic Campus, EN-80, Sector-V, Salt Lake City, Kolkata, India; University of California San Francisco, UNITED STATES

## Abstract

*Candida albicans* is a commensal of the human microbiota that can form biofilms on implanted medical devices. These biofilms are tolerant to antifungals and to the host immune system. To identify novel genes modulating *C*. *albicans* biofilm formation, we performed a large-scale screen with 2,454 *C*. *albicans* doxycycline-dependent overexpression strains and identified 16 genes whose overexpression significantly hampered biofilm formation. Among those, overexpression of the *ZCF15* and *ZCF26* paralogs that encode transcription factors and have orthologs only in biofilm-forming species of the *Candida* clade, caused impaired biofilm formation both in vitro and in vivo. Interestingly, overexpression of *ZCF15* impeded biofilm formation without any defect in hyphal growth. Transcript profiling, transcription factor binding, and phenotypic microarray analyses conducted upon overexpression of *ZCF15* and *ZCF26* demonstrated their role in reprogramming cellular metabolism by regulating central metabolism including glyoxylate and tricarboxylic acid cycle genes. Taken together, this study has identified a new set of biofilm regulators, including *ZCF15* and *ZCF26*, that appear to control biofilm development through their specific role in metabolic remodeling.

## Abstract 2

Fungal biofilms are complex communities that can form on surfaces of implanted medical devices. This study uses an overexpression screen to identify novel modulators of Candida albicans biofilm formation, revealing that the transcription factors ZCF15 and ZCF26 regulate metabolic reprogramming during the transition from planktonic to biofilm growth.

## Introduction

*Candida albicans* is a commensal of the human microbiota that resides on the mucosal surfaces of the gastrointestinal and genital tracts. Under certain circumstances, such as if epithelial barriers are disturbed or the immune system is impaired, the fungus undergoes a transition from commensalism to pathogenicity [1]. This transition is well regulated by both the host immune system and fungal-specific virulence attributes.

*C*. *albicans* can form biofilms, which represent a major fungal virulence attribute [2,3]. Biofilms are microbial communities attached to surfaces and protected by self-produced extracellular substances [4]. Cells in a biofilm are more adherent and more tolerant to antimicrobials as compared to the free-floating planktonic cells and these properties make biofilm-associated infections a clinical challenge [2,5,6]. *C*. *albicans* biofilms are structured and composed of differentiated cell types encased in an extracellular matrix. Briefly, the *C*. *albicans* biofilm developmental process involves the attachment of yeast cells to a surface and their proliferation to establish a basal layer. Basal layer cells undergo cellular differentiation in multiple cell types including hyphae and pseudo-hyphae that become encased in a self-produced extracellular matrix, leading to a mature biofilm [4,7]. These biofilms can be the source of disseminated infections that can, in turn, lead to invasive systemic infections of tissues and organs [3,8,9].

Among the *Candida* clade, only a few species closely related to *C*. *albicans*, namely *Candida dubliniensis* and *Candida tropicalis*, can form a complex biofilm. The less closely related species *Candida parapsilosis*, *Loderomyces elongisporus*, and *Spathaspora passalidarum* are also able to form biofilms, but these are structurally different and of lesser biomass than those of *C*. *albicans* [10–14]. Transcript profiling, proteome analyses and metabolomic studies of *C*. *albicans* planktonic and biofilm cells have shown that cellular differentiation and metabolic reprogramming are 2 critical events that occur when *C*. *albicans* cells transition from the planktonic to the biofilm growth mode [13,15–21]. Studies on *C*. *albicans* transcription regulators have suggested that a well-coordinated crosstalk operates during biofilm development. For instance, transcription regulators, Ace2, Brg1, Efg1, Ndt80, Mss11, Tec1, Flo8, Rob1, and Ume6 are essential for *C*. *albicans* hyphal development and are also needed for *C*. *albicans* biofilm formation [13,22,23]. In parallel, Tye7 regulates the glycolytic flux, and the lack of this transcription factor leads to impaired biofilm formation [15]. In addition, amino acid metabolism is modulated during biofilm formation, and it has been shown that the Gcn4 regulator of the amino acid biosynthetic pathways is important for efficient biofilm formation [19]. Yet, it is notable that most modulators in the regulation of *C*. *albicans* biofilm formation identified so far are positive regulators. Only a few transcription regulators such as Nrg1, Zcf32, and Upc2 have been shown to play a negative role during *C*. *albicans* biofilm formation [18,24,25]. This may be a consequence of the approach used to identify these modulators, as the biofilm growth conditions did not allow an increase in biofilm biomass to be observed when the target genes were inactivated, as expected for genes encoding negative regulators of biofilm formation [18]. In this study, we sought to identify additional negative regulators of biofilm formation and reasoned that their overexpression would result in reduced biofilm biomass in a biofilm formation assay.

Large collections of *C*. *albicans* overexpression strains are becoming available and have proven useful to identify genes with a role in *C*. *albicans* morphogenesis, genome plasticity, biofilm formation, antifungal tolerance, and intestinal colonization [26–30]. Using a novel collection of 2,454 *C*. *albicans* doxycycline-dependent overexpression strains derived from the *C*. *albicans* ORFeome [27,28], we could identify 16 genes whose overexpression led to reduced biofilm formation. Among these genes, the *ZCF15* and *ZCF26* paralogs encode zinc cluster transcription factors whose overexpression leads to impaired biofilm growth in vitro and in vivo. Transcript profiling and ChIP-sequencing analyses demonstrated that both *ZCF15* and *ZCF26* directly regulate the expression of genes associated with cellular metabolism, including the genes of the glycolysis, glyoxylate cycle, and tricarboxylic acid (TCA) cycle, known to be differentially expressed when *C*. *albicans* proliferates as biofilms. Altogether, we discovered novel transcription regulators that recently appeared to regulate metabolic remodeling during planktonic to biofilm transition.

## Results

### A large-scale overexpression screen identifies *C*. *albicans* negative regulators of biofilm formation

**Fig 1:**
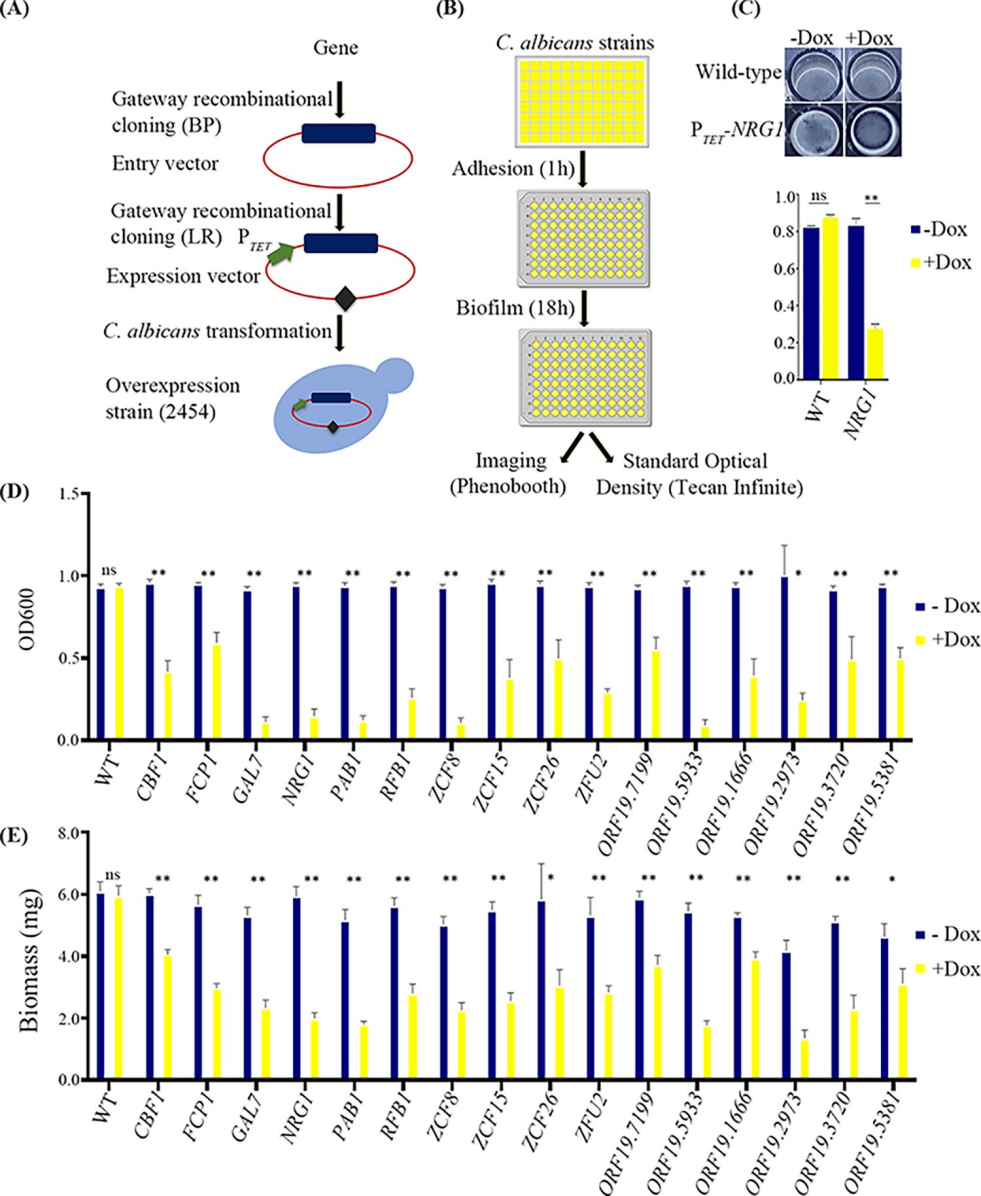
High-throughput screen for biofilm formation with *C*. *albicans* overexpression strains. (**A**) Schematic showing the construction of 2,454 *C*. *albicans* P_*TET*_ overexpression strains. (**B**) Overview of the in vitro screening strategy for the collection of *C*. *albicans* overexpression strain for biofilm formation. Cells were grown overnight in 96-deep-well plates in YPD with or without 25 μg/ml doxycycline. Then, 0.2 μl of culture was diluted in 200 μl of YPD medium with or without 25 μg/ml of doxycycline and transferred to FBS pre-coated 96-well polystyrene plates that were incubated at 37°C for 1 h for adhesion to occur. Then, the medium was aspirated, and the wells were washed with 1× PBS. A fresh aliquot of 200 μl of YPD medium with or without 25 μg/ml of doxycycline was added and biofilms were allowed to develop for 18 h at 37°C at 110 rpm. After 18 h, the medium was discarded, the wells washed with 1× PBS and photographed. Quantification of biofilms was determined by measuring the standard optical density using a Tecan infinite M200. Created with BioRender.com. (**C**) Biofilm formation by *C*. *albicans* wild-type and P_*TET*_*-NRG1* overexpression strains with or without doxycycline. (**D** and **E**) *C*. *albicans* overexpression strains identified in the screen and the WT control were grown overnight in YPD medium, with or without 25 μg/ml doxycycline. Biofilms were allowed to develop in 96-well polystyrene plates (**D**) or in 12-well polystyrene plates (**E**) in YPD medium with or without 25 μg/ml doxycycline at 37°C for 18 h. (**D**) Standard optical density was measured to quantify the extent of biofilm formation using a Tecan infinite M200. (**E**) Dry weight biomass of biofilms formed by the wild-type and the overexpression strains. Gene names are given below the bar. Statistical significance was determined using Holm–Sidak method by performing multiple *t* test between uninduced and induced condition datasets. ns: *P* > 0.05; *: P ≤ 0.05; **: *P* ≤ 0.01. The data underlying this figure can be found in S2 Data (C), S3 Data (D), and S4 Data (E). FBS, fetal bovine serum.

In the frame of the *C*. *albicans* ORFeome project, 5,099 ORFs representing approximately 83% of *C*. *albicans* predicted ORFs were cloned into a Gateway donor vector [28]. A total of 2,454 of these ORFs were then transferred in a tetracycline-dependent overexpression vector and introduced into a suitably engineered *C*. *albicans* strain [27] (**Fig 1A**). This unbiased *C*. *albicans* overexpression collection was used to uncover genes whose overexpression hampers *C*. *albicans* biofilm formation.

We first set up the experimental conditions allowing the detection of genes whose overexpression would alter biofilm formation as compared to either the uninduced condition or the wild-type control. A strain overexpressing *NRG1*, a known negative transcription regulator of *C*. *albicans* morphogenesis and biofilm formation [25], was used to optimize the screening conditions. The wild-type control strain (CEC4665) and a P_*TET*_*-NRG1* overexpression strain (CEC6039) were induced to form biofilms in 96-well polystyrene plates at 37°C for 18 h in YPD medium, with or without 25 μg/ml doxycycline (**Fig 1B**). The extent of biofilm formation was assessed by plate imaging and by quantifying standard optical density [31]. In these conditions, overexpression of *NRG1* led to decreased biofilm formation as compared to either the uninduced condition or the wild-type control (**Fig 1C**).

Then, the conditions optimized with the *NRG1* overexpression strain were individually applied to the 2,454 doxycycline-dependent *C*. *albicans* overexpression strains. We identified 16 candidate genes that, when overexpressed, inhibited biofilm growth as compared to either wild-type or uninduced cells (**S1A Fig**). These genes encode transcription factors (*CBF1*, *NRG1*, *RBF1*, *ZFU2*, *ZCF8*, *ZCF15*, and *ZCF26*), a protein phosphatase (*FCP1*), nucleic acid-binding proteins (*PAB1*, *ORF19*.*2973*, and *ORF19*.*5381*), or uncharacterized ORFs (*ORF19*.*1666*, *ORF19*.*3720*, *ORF19*.*5933*, *ORF19*.*7199*, and *GAL7*). Of note, our large-scale screen identified Nrg1 as a negative regulator of biofilm formation.

To confirm that overexpression of the 16 identified genes genuinely hampered biofilm formation, independent overexpression strains for these genes were constructed and tested for their ability to form biofilms in the presence or absence of doxycycline. We could confirm the observed phenotypes for all candidate genes (**Fig 1D**). We also confirmed the reduction of biofilm formation upon overexpression of this set of genes by measuring the dry weight biomass produced on the surface of polystyrene plates (**Fig 1E**). To test whether the reduction in biofilm formation could be the result of a general growth defect upon overexpression, a growth assay was performed with the wild-type control and the 16 overexpression strains, with or without doxycycline. In total, 14 out of the 16 mutants showed no significant alteration in their doubling time upon induction (**S1B Fig**). Conversely, *PAB1* overexpression resulted in approximately 1.5-fold increase in doubling time as compared to both the wild-type strain or uninduced conditions, and the *ORF19*.*5381* overexpression strain grew poorly in the presence of doxycycline (approximately 1.9-fold increase in doubling time as compared to uninduced condition and approximately 4-fold increase in doubling time as compared to the wild-type control) (**S1B Fig**). Of note, although the *ORF19*.*5381* overexpression strain grew poorly in the absence of doxycycline (approximately 2.2-fold increase in doubling time as compared to the uninduced wild-type control), it could form robust biofilms in these conditions (**S1A Fig**). Therefore, we did not investigate the *PAB1* and *ORF19*.*5381* genes further. The 14 remaining candidate genes did not cause any significant alteration of growth between uninduced and induced conditions, indicating a direct role in biofilm formation. We decided to focus on genes encoding transcription factors, namely *NRG1*, *RBF1*, *ZFU2*, *ZCF8*, *ZCF15*, and *ZCF26*. *CBF1* was excluded from further studies as it is a characterized transcription factor that binds to the ribosomal protein gene promoters and whose knock-out mutant exhibits a slow growth phenotype [32].

**Fig 2:**
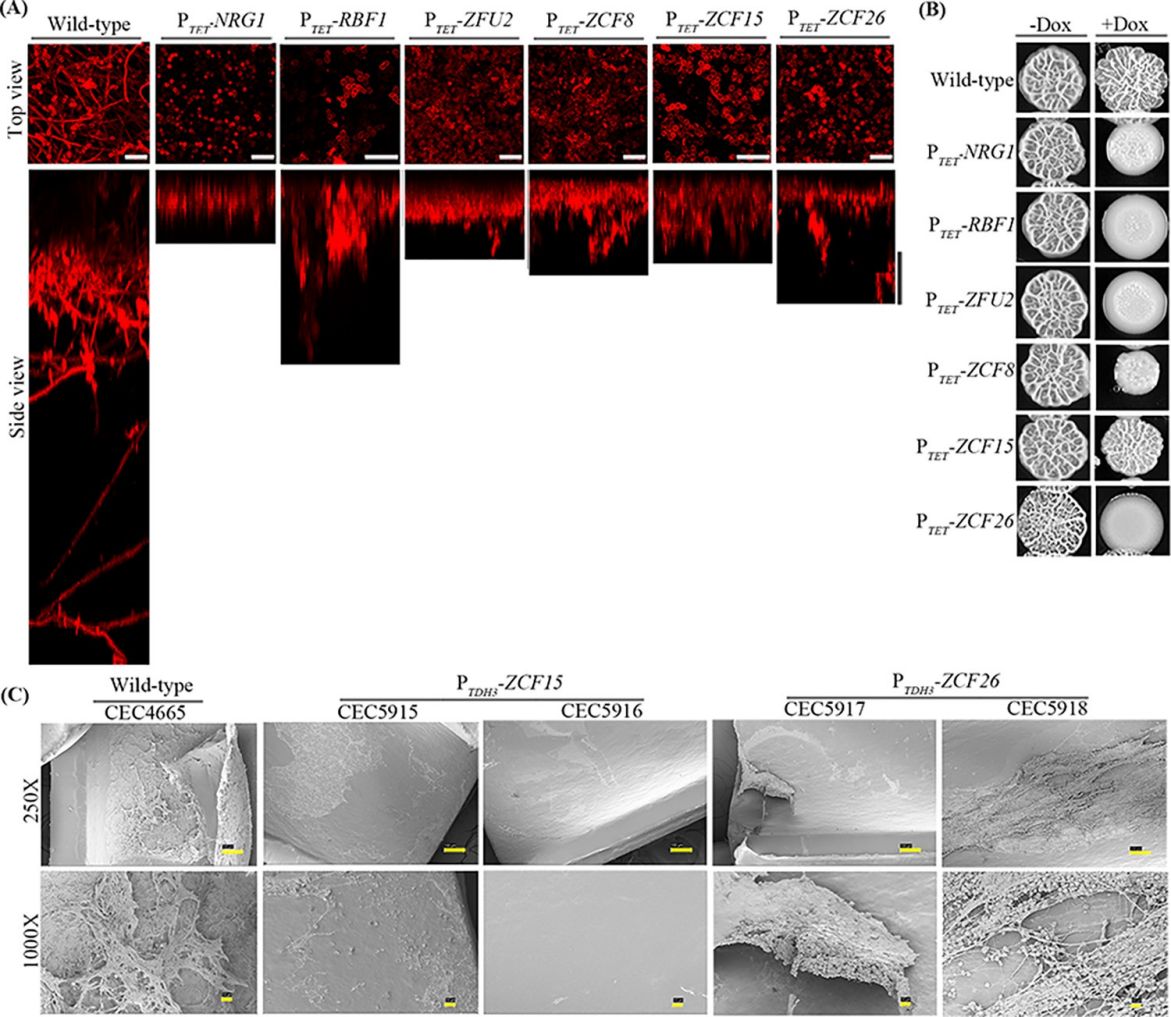
Overexpression of *ZCF15* and *ZCF26* leads to a rudimentary biofilm in rat catheter in vivo model. (**A**) Wild-type (CEC4665) and overexpression strains P_*TET*_*-NRG1* (CEC6039), P_*TET*_*-RBF1* (CEC6043), P_*TET*_*-ZFU2* (CEC6044), P_*TET*_*-ZCF8* (CEC6053), P_*TET*_*-ZCF15* (CEC6052), and P_*TET*_*-ZCF26* (CEC6051) were allowed to adhere to silicone squares in 12-well polystyrene plates in YPD medium supplemented with 25 μg/ml doxycycline at 37°C for 1 h. Biofilms were allowed to grow for 18 h at 110 rpm and stained with concanavalin A-Alexa Fluor 594 conjugate for 2 h. Biofilms were imaged by CLSM. Images are projections of the top and side views. Representative images of at least 3 replicates are shown. Scale bars for both top view and side view: 25 μm. (**B**) The extent of filamentation of wild-type, P_*TET*_*-NRG1*, P_*TET*_*-RBF1*, P_*TET*_*-ZFU2*, P_*TET*_*-ZCF8*, P_*TET*_*-ZCF15*, and P_*TET*_*-ZCF26* strains was estimated by spot assay on YPD agar containing 20% FBS with or without 25 μg/ml doxycycline and 3 days of incubation at 37°C. (**C**) In vivo biofilm formation assay was performed using the rat catheter model. Wild-type (CEC4665), P_*TDH3*_*-ZCF15* (CEC5915 and CEC5916) and P_*TDH3*_*-ZCF26* (CEC5917 and CEC5918) strains were inoculated in a rat intravenous catheter and were allowed to form biofilms for 24 h. Then, biofilms were visualized using SEM. The images are 250× and 1,000× magnification views of the catheter lumens. The scale bar for 250× magnification is 100 μm and 10 μm for 1,000× magnification. CLSM, confocal laser scanning microscopy; FBS, fetal bovine serum.

To get further insight in the role of the 6 regulators in biofilm formation, we first determined the structure and thickness of biofilms formed upon their overexpression by performing confocal laser scanning microscopy (CLSM) with biofilms grown on silicone squares in 12-well polystyrene plates at 37°C for 18 h in YPD medium in the presence of doxycycline [22]. CLSM analysis revealed that biofilms formed upon overexpression of the 6 candidate genes were mostly composed of yeast cells (**Fig 2A**, top view) resulting in a reduction in the biofilm thickness as compared to the wild-type control strain (**Fig 2A**, side view). These results further confirmed that overexpression of *NRG1*, *RBF1*, *ZFU2*, *ZCF8*, *ZCF15*, and *ZCF26* leads to impaired biofilm production.

### Filamentation is unaffected upon *ZCF15* overexpression

To test whether the defect in biofilm formation upon overexpression of the 6 transcription factor genes is merely the consequence of a defect in hyphal growth, their filamentation was examined by spot assays on solid YPD medium containing 20% fetal bovine serum (FBS) with or without 25 μg/ml doxycycline (**Fig 2B**). In these conditions, overexpression strains were forming smooth colonies (*RBF1*, *ZFU2*, and *ZCF26*) or exhibited reduced wrinkling (*NRG1* and *ZCF8*), as compared to the wild-type or uninduced conditions. Interestingly, cells overexpressing *ZCF15* still formed wrinkled colonies. We further examined the colony phenotype of the overexpression strains at the single colony level. *NRG1*, *RBF1*, *ZFU2*, and *ZCF26* overexpression led to a defect in colony wrinkling. In contrast, *ZCF8* and *ZCF15*-overexpressing cells were able to form wrinkled colonies (**S2A Fig**). We also inspected the extent of hyphal formation in liquid YPD medium containing 20% FBS with or without doxycycline. In these conditions, strains overexpressing *NRG1*, *RBF1*, and *ZCF26* were compromised for their ability to form hyphae as compared to wild-type control or the uninduced condition. However, overexpression of *ZCF8*, *ZFU2*, and *ZCF15* did not prevent hyphal formation in liquid medium (**S2B Fig)**. In conclusion, these results indicate that the biofilm formation defect observed upon *ZCF15* overexpression is not the result of impaired hyphal growth under hyphae inducing conditions.

### Evolutionary appearance of *C*. *albicans* transcription factors identified by overexpression approaches

In this study, we identified transcription regulators whose overexpression caused a reduction in biofilm formation. Therefore, we questioned the conservation of these novel biofilm regulators in different *Candida* species. To this aim, a search for orthologs of *NRG1*, *RBF1*, *ZCF8*, *ZCF15*, *ZCF26*, and *ZFU2* was performed in the Saccharomycetes using PSI-BLAST and Hidden Markov models [33]. Further, the presence of orthologs of these transcription factor genes in closely related species was evaluated using reciprocal best hits (RBH) analysis. The repressor of morphogenesis Nrg1 is present in most sequenced species of the Saccharomycetes, including *Saccharomyces cerevisiae*. Rbf1, another repressor of filamentation in *C*. *albicans* is present in other members of the CTG clade (i.e., species in which the CUG codon encodes serine instead of a universal leucine) and Zcf8, a regulator of vacuolar function [34], is present only in a few species of the CTG clade [35]. Interestingly, the 3 other regulators, namely Zfu2, Zcf15, and Zcf26 are restricted to CTG clade species able to form biofilms. Transcription factor Zcf15 is found in *C*. *albicans*, *C*. *dubliniensis*, *C*. *tropicalis*, and *C*. *parapsilosis*, Zcf26 in *C*. *albicans*, *C*. *dubliniensis*, *C*. *tropicalis*, *C*. *parapsilosis*, *L*. *elongisporus*, and *S*. *passalidarum* and Zfu2 occurs in *C*. *albicans* and *C*. *dubliniensis* (**S2C Fig**). We also examined the phylogenetic relationship between the transcription factors identified in this study. Phylogenetic analyses suggested that *ZCF15* and *ZCF26* are paralogs and that *ZCF15* originates from a duplication of the *ZCF26* gene (**S2D Fig**). Our phylogenetic analysis confirms the published *ZCF15* and *ZCF26* phylogenetic relationship [36]. Interestingly, *C*. *albicans* possesses a third paralogous gene, *ZCF25*, also restricted to the CTG clade (**S2D Fig**). Of these 3 paralogous genes, *ZCF15* and *ZCF26* show greater amino acid sequence similarity (63.73% identity between Zcf15 and Zcf26 versus 34.21% between Zcf15 and Zcf25, or 38.16% between Zcf26 and Zcf25). Of note, the C-terminus parts of the transcription factors show lower amino acid sequence conservation (**S3A Fig**). As the *ZCF25* OE strain was not present in the OE collection used for the screen, we constructed the strain and performed biofilm and filamentation assays; unlike *ZCF15* or *ZCF26*, the overexpression of *ZCF25* did not lead to any significant difference compared to the wild type in its ability to form biofilms or filaments (**S3B and S3C Fig**). In conclusion, these analyses revealed a recent appearance of transcription factors Zcf15, Zcf26, and Zfu2 only in CTG clade species that form biofilms.

### Overexpression of *ZCF15* and *ZCF26* leads to impaired in vivo biofilm formation

We decided to focus on the role of the 2 paralogs *ZCF15* and *ZCF26* in biofilm formation. To rule out that the reduction in biofilm formation upon overexpression of *ZCF15* and *ZCF26* is linked to our experimental conditions, namely the use of YPD medium, we examined the biofilm-forming behavior of strains overexpressing *ZCF15* and *ZCF26* using a well-established Spider medium biofilm formation model. Similar to the results observed in our screen, the overexpression of *ZCF15* and *ZCF26* resulted in impaired biofilm formation when the biofilm assay was performed in Spider medium (**S4A Fig**).

Next, to assess whether the results observed upon in vitro biofilm formation could be recapitulated in vivo, we placed *ZCF15* and *ZCF26* under the control of the constitutive *TDH3* promoter. We examined the constitutive *ZCF15-* and *ZCF26-*overexpression strains for their ability to produce biofilms and filaments in vitro. Similar to conditional overexpression, constitutive overexpression of *ZCF15* and *ZCF26* resulted in impaired biofilm formation in vitro and overexpression of *ZCF26* alone resulted in impaired filamentation (**S4B** and **S4C Fig**). To investigate the impact of *ZCF15* and *ZCF26* overexpression on *C*. *albicans* biofilm formation in vivo, we used a well-established rat-catheter model [37]. Catheters were inoculated intraluminally with the wild-type control (CEC4665) and 2 independent clones of P_*TDH3*_*-ZCF15* (CEC5915 and CEC5916) and P_*TDH3-*_*ZCF26* (CEC5917 and CEC5918) overexpression strains. After 24 h of biofilm growth, the catheters were removed, and the luminal surfaces of the catheters were imaged by scanning electron microscopy (SEM). Overexpression of *ZCF15* failed to produce any biofilm on rat catheters, whereas overexpression of *ZCF26* resulted in less robust biofilm formation than the wild-type strain (**Fig 2C**). These in vivo results thus confirmed the role of transcription factors Zcf15 and Zcf26 in modulating *C*. *albicans* biofilm formation in vitro and in vivo.

### Transcriptome alterations upon Zcf15 and Zcf26 overexpression

To understand the mechanisms by which Zcf15 and Zcf26 overexpression inhibits *C*. *albicans* biofilm formation and to uncover the gene circuitry they orchestrate, we conducted a genome-wide transcript profiling with P_*TET*_-*ZCF15* (CEC6052), P_*TET*_*-ZCF26* (CEC6051), and the wild-type control strain (CEC4665) by RNA sequencing under conditions of biofilm formation in the presence of 25 μg/ml doxycycline. To rule out an effect of doxycycline on the overall gene expression profile, we also performed transcript profiling with the wild-type strain grown under biofilm conditions with or without doxycycline (**S1A Data**). In the latter experiment, we considered as differentially expressed those genes that showed a change in expression level by Log2>1.2 or Log2<-1.2 and *p* < 0.05 in response to doxycycline addition. Transcript profiling of *C*. *albicans* wild-type cells exposed to doxycycline revealed the upregulation of 1 gene and down-regulation of 14 genes as compared with untreated wild-type cells (**S1B and S1C Data**). Genes whose expression levels were altered by the presence of doxycycline in wild-type cells were excluded from transcriptome analysis of strains overexpressing *ZCF15* and *ZCF26*. RNA expression analysis with P_*TET*_*-ZCF15* and P_*TET*_-*ZCF26* overexpression strains displayed differential expression of 923 and 1,239 genes, respectively, when Log2>1.2 or Log2<-1.2 and *p* < 0.05 were used as the thresholds for differentially expressed genes as compared to the doxycycline-exposed wild-type strain (**S1D and S1E Data**). Overexpression of *ZCF15* resulted in the up-regulation of 406 coding genes and the down-regulation of 517 genes as compared to the wild-type control (**S1F and S1G Data**). Similarly, when *ZCF26* was overexpressed, 552 genes were up-regulated, and 687 genes were down-regulated (**S1H and S1I Data**). Interestingly, comparison of differentially expressed genes upon overexpression of *ZCF15* or *ZCF26* showed a common set of 221 up-regulated genes and 410 down-regulated genes (**S5A and S5B Fig**).

**Fig 3:**
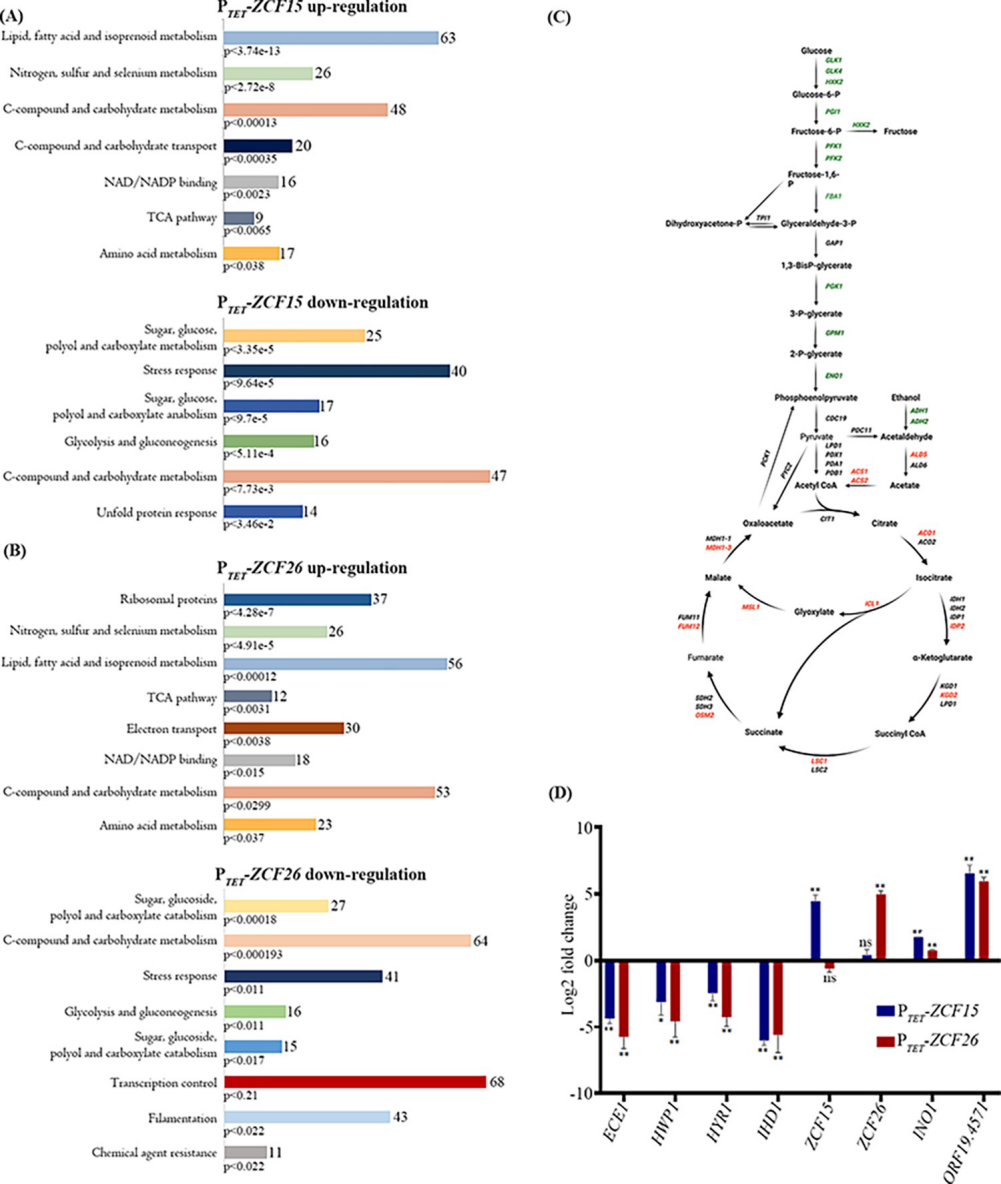
Transcript profiling of *C*. *albicans* transcription factors *ZCF15* and *ZCF26* during biofilm mode of growth. RNA expression profiling of P_*TET*_*-ZCF15* (**A**) and P_*TET*_*-ZCF26* (**B**) strains grown in biofilm condition with 25 μg/ml doxycycline was performed. Functional classification of genome-wide up- and down-regulated genes upon overexpression of *ZCF15* or *ZCF26* was determined using FungiFun2 and statistically significant altered categories are shown. (**C**) Central metabolic pathways of *C*. *albicans* is illustrated to show the genes of the glycolytic, glyoxylate, and TCA pathways altered when *ZCF15* and *ZCF26* overexpressing strains are grown with 25 μg/ml doxycycline in biofilm-forming conditions. Down-regulated genes are indicated in green, up-regulated genes in red and nonsignificantly altered genes in black. Created with BioRender.com. (**D**) qPCR analysis was performed to validate the altered expression of biofilm-related genes with wild-type parental cells, P_*TET*_*-ZCF15* and P_*TET*_*-ZCF26* overexpression strains grown in biofilm conditions in the presence of doxycycline. ΔC_T_ values were derived after normalizing the expression of genes of interest with that of *TEF3*, and ΔΔC_T_ values were calculated for the relative expression of the indicated genes. Statistical significance was determined using Holm–Sidak method by performing multiple *t* tests; ns: *P* > 0.05; *: *P* ≤ 0.05; **: *P* ≤ 0.01. The data underlying this figure can be found in S5 Data. TCA, tricarboxylic acid.

To examine the altered pathways upon overexpression of the transcription factors Zcf15 and Zcf26, the differentially expressed genes were categorized into different functional classes using FungiFun 2 [38]. This analysis revealed that genes belonging to cellular metabolism (lipid, fatty acid and isoprenoid, amino acid, C-compound and carbohydrate, nitrogen, sulfur, and selenium metabolism), the TCA pathway, NAD/NADP binding, and cellular transport were up-regulated when *ZCF15* was overexpressed. Categories significantly down-regulated included sugar, glucose, polyol and carboxylate metabolism, C-compound and carbohydrate metabolism, stress response, glycolysis, and gluconeogenesis (**Fig 3A**).

Similarly, cellular metabolism (lipid, fatty acid and isoprenoid, amino acid, C-compound and carbohydrate and nitrogen, sulfur, and selenium metabolism), the TCA pathway, protein synthesis (ribosomal proteins), NAD/NADP binding, and electron transport were the categories significantly up-regulated when *ZCF26* was overexpressed, while sugar, glucose, polyol and carboxylate metabolism, C-compound and carbohydrate metabolism, stress response, glycolysis and gluconeogenesis, filamentation, and transcription control were the categories significantly down-regulated (**Fig 3B**). Importantly, genes relevant to *C*. *albicans* morphogenesis including *ACE2*, *BRG1*, *CPH2*, *EFG1*, *FKH2*, *ASH1*, *RAS1*, etc. were down-regulated when *ZCF26* was overexpressed, whereas no significant alterations in the expression of these genes were found when *ZCF15* was overexpressed, suggesting that Zcf26 plays a role in the regulation of both morphogenesis and biofilm formation.

Since transcript profiling pinpointed an alteration of central metabolic pathways of *C*. *albicans*, we specifically examined expression of glycolysis and TCA cycle genes. Interestingly, we noticed that overexpression of *ZCF15* and *ZCF26* severely hampered the expression of glycolysis genes known to be up-regulated during *C*. *albicans* biofilm formation [15]. In contrast, genes of the glyoxylate shunt and TCA cycle were up-regulated upon overexpression of *ZCF15* and *ZCF26* (**Figs 3C** and **S5C**). In addition, several critical biofilm-associated genes that are up-regulated during *C*. *albicans* biofilm formation, such as *HWP1*, *ECE1*, *HYR1*, *HSP104*, and *IHD1* were down-regulated upon overexpression of *ZCF15* and *ZCF26*. Similarly, biofilm-repressed genes such as *INO1* and *ORF19*.*4571* were up-regulated when *ZCF15* and *ZCF26* were overexpressed. These subsets of biofilm-critical genes were also validated by quantitative real-time PCR (**Fig 3D**).

In conclusion, global gene expression analyses with strains overexpressing the transcription factors Zcf15 or Zcf26 suggested a role in metabolic reprogramming during *C*. *albicans* biofilm development.

### Identification of directly bound targets of Zcf15 and Zcf26 by ChIP-sequencing

**Fig 4:**
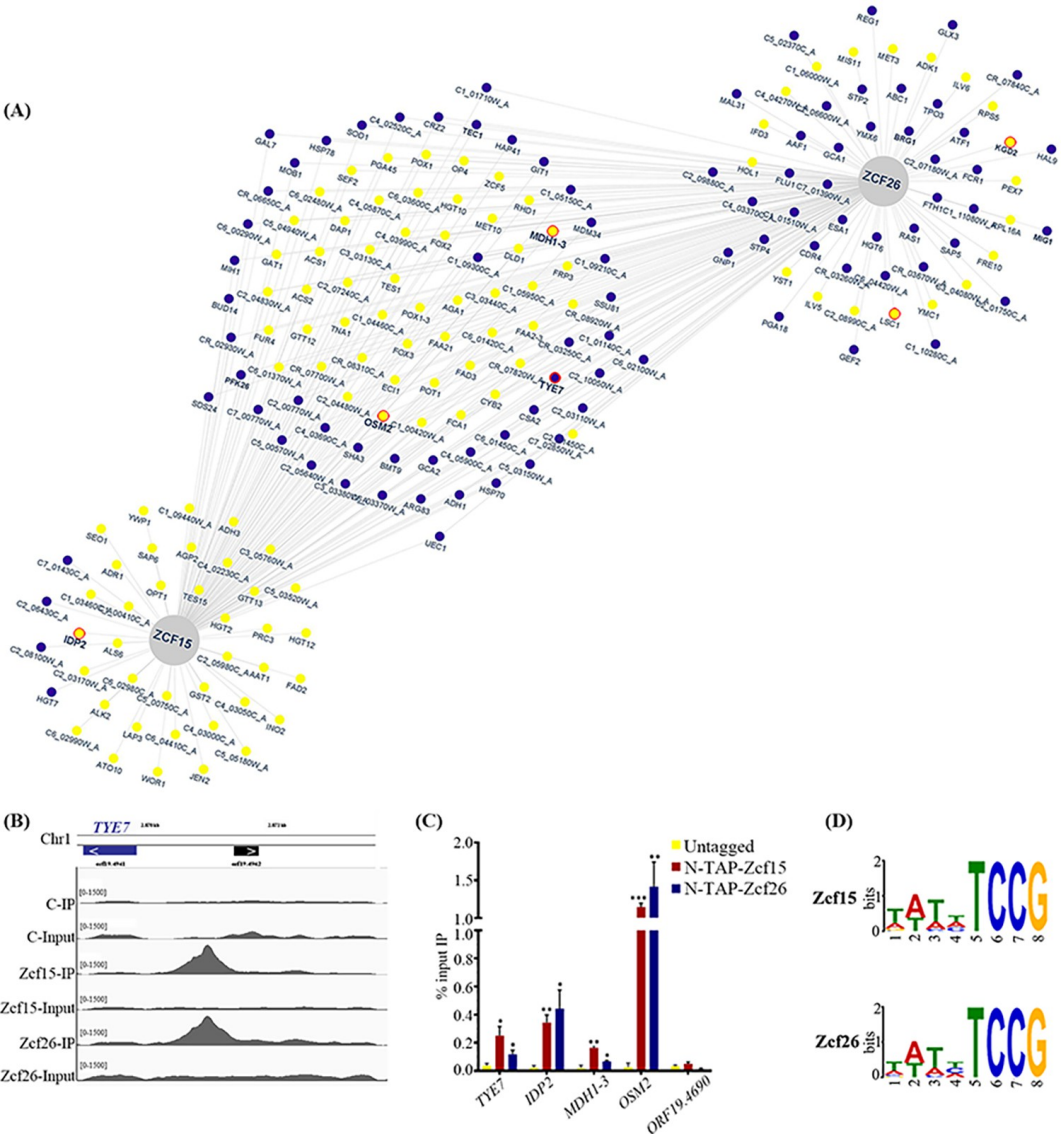
Binding of transcription factors Zcf15 and Zcf26 to the *C*. *albicans* genome. The DNA binding profile of Zcf15 and Zcf26 obtained by ChIP-sequencing was compared with gene expression data obtained from strains overexpressing *ZCF15* or *ZCF26*. (**A**) Network view for Zcf15 and Zcf26. Genes regulated and bound by Zcf15 (left), Zcf26 (right), or both (middle) are indicated in yellow (up-regulation) or blue (down-regulation). The interaction network was generated using Cytoscape [73]. Genes further analyzed in (**C**) are circle in red. (**B**) Binding of Zcf15 (middle lanes) and Zcf26 (bottom lanes) at the promoter of *TYE7*, a transcription factor that regulates the expression of genes of the glycolytic pathway. (**C**) ChIP assays were performed on wild-type untagged, *N-TAP-ZCF15* and *N-TAP-ZCF26* strains. Immunoprecipitated (IP) DNA fractions were analyzed by qPCR with primer pairs specific for *TYE7*, *IDP2*, *MDH1*-3, and *OSM2* promoter regions (see **S2 Table**); *ORF19*.*4690* was used as a negative control. Quantitative RT-PCR was performed with untagged strain samples to detect the background DNA elution in the ChIP assay. The enrichment of Zcf15 and Zcf26 to the promoters of indicated genes is represented as a percent input immunoprecipitated with standard error of mean. The values from 3 independent ChIP experiments were plotted. Statistical significance was determined using Holm–Sidak method by performing multiple *t* test. *: *P* ≤ 0.05; **P ≤ 0.01; ***: *P* ≤ 0.001. (**D**) Genome-wide binding motifs of Zcf15 and Zcf26 were identified using MEME-ChIP. The data underlying this figure can be found in S6 Data. ChIP, chromatin immunoprecipitation.

Genes directly regulated by Zcf15 and Zcf26 were identified by chromatin immunoprecipitation followed by high-throughput sequencing (ChIP-seq), which allowed us to map the binding sites of the regulators in the *C*. *albicans* genome. To implement the ChIP assay, we fused the N-terminus of the transcription factors Zcf15 or Zcf26 with a tandem affinity purification (TAP) epitope tag. The functionality of TAP-Zcf15 and TAP-Zcf26 was verified by testing the impact of their overexpression on biofilm formation and filamentation. Overexpression of *TAP-ZCF15* or *TAP-ZCF26* phenocopied overexpression of *ZCF15* or *ZCF26*, respectively (**S6A and S6B Fig**). We then performed a ChIP assay followed by Illumina sequencing using an untagged *C*. *albicans* control strain and 2 independent clones of TAP-tagged Zcf15 and Zcf26 strains growing as biofilms. We detected the binding of Zcf15 and Zcf26 in 317 and 363 intergenic regions of the *C*. *albicans* genome, respectively. Among these regions, we then identified bona fide promoter regions and uncovered that Zcf15 binds to the promoters of 431 ORFs, whereas Zcf26 binds to the promoters of 494 ORFs (**S1J and S1K Data**). We then compared the results of transcript profiling and ChIP-seq to identify directly regulated genes. Zcf15 binds to the promoters of 89 up-regulated and 43 down-regulated genes. Similarly, Zcf26 binds to the promoters of 70 up-regulated and 87 down-regulated genes. A comparison of all genes directly bound either by Zcf15 or Zcf26 and differentially expressed upon their overexpression showed an overlap of 51 up-regulated and 41 down-regulated genes (**Fig 4A and S1L Data**). Strikingly, both Zcf15 and Zcf26 bind to the promoter of the glyoxylate and the TCA cycle genes, namely *IDP2*, *KGD2*, *MDH1-3*, and *OSM2*. Moreover, both regulators also bind to the promoters of genes of the glycolytic pathway such as *TYE7*, encoding a transcription factor and *PFK26* that encodes a 6-phosphofructo-2-kinase. The binding of Zcf15 and Zcf26 to the promoters of a subset of genes was further verified by ChIP-quantitative PCR (ChIP-qPCR). Promoter region of *ORF19*.*4690* was used as a control since it is not bound either by Zcf15, Zcf26, or master regulators of biofilm formation (**Fig 4C**). Then, based on genome-wide binding events of Zcf15 and Zcf26, we determined their binding motif using MEME-ChIP [39]. Since Zcf15 and Zcf26 share many targets in which their binding area overlap, the motif identified here for Zcf15 and Zcf26 was very similar (WWWHTCCG) (**Fig 4D**) confirming their common evolutionary origin.

### Metabolic profiling of *ZCF15* and *ZCF26* overexpression strains by phenotypic microarrays

**Fig 5:**
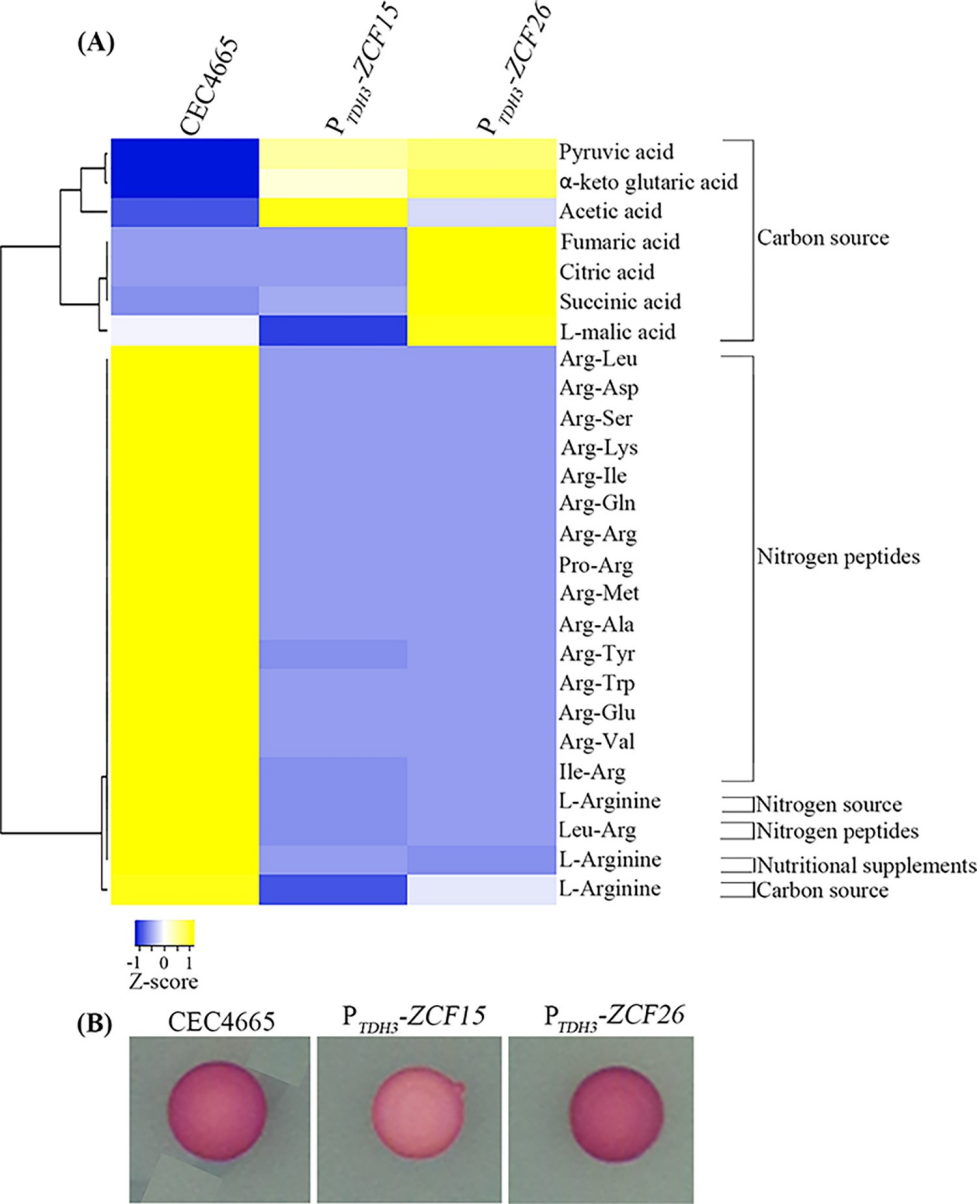
Metabolic activities profile of transcription factors *ZCF15* and *ZCF26*. **(A)** Comparison of metabolic activities of parental reference strain and *ZCF15* and *ZCF26* overexpression strains is shown as a heat-map. Metabolic activities were monitored at 30°C for 96 h and were measured using the AUC. Metabolic activity in the indicated growth conditions is represented on a scale from −1 (minimum growth, blue) to +1 (maximum growth, yellow). **(B)** Wild-type, P_*TDH3*_*-ZCF15* and P_*TDH3*_*-ZCF26* strains were spotted on YPD solid medium and allowed to grow at 30°C for 24 h. Cells were then covered with a 0.025% TTC-agarose solution and pictures were taken after a 30-min incubation at room temperature. The data underlying this figure can be found in S7 Data. AUC, area under the curve; TTC, triphenyltetrazoliumchloride.

Both transcript profiling and ChIP-sequencing highlighted the role of Zcf15 and Zcf26 in controlling metabolic remodeling specifically the genes of the glyoxylate and the TCA cycles during *C*. *albicans* biofilm formation. Therefore, we examined the metabolic profiles of *ZCF15* and *ZCF26* overexpression strains under different growth conditions. To this aim, we performed phenotypic microarrays (PM), a high-throughput tool to get the global metabolic profiles of microbial cells [40,41]. The growth of the wild-type and of the constitutive overexpression strains P_*TDH3*_*-ZCF15* and P_*TDH3*_*-ZCF26* were examined in PM plates (Biolog) coated with different nutrients and chemical substances: carbon sources (PM01 and PM02), nitrogen source (PM03), nutritional supplements (PM05), and nitrogen peptides (PM06 and PM08). The global growth profile of the strains was monitored at 30°C for 96 h and is represented as heat-map (**S7 Fig**). These PM-based results revealed an enhanced growth of strains overexpressing *ZCF15* and *ZCF26* when succinic acid, acetic acid, α-keto-glutaric acid, and pyruvic acid were used as carbon sources. In addition, we noticed that strains overexpressing *ZCF15* and *ZCF26* showed a reduced growth when L-arginine was used either as a carbon source, a nitrogen source, or provided as a nutrient supplement. Moreover, both *ZCF15* and *ZCF26* overexpression strains displayed a reduced growth when di-peptides containing Arg residues (Arg-Glu, Arg-Gln, Arg-Ile, Arg-Met, Ile-Arg, Arg-Lys, Arg-Asp, Arg-Leu, Arg-Ser, Arg-Val, Arg-Trp, Arg-Arg, Pro-Arg, Arg-Tyr, Leu-Arg, Arg-Ala) were used as nitrogen source (**Fig 5A**). These results coincide with the previous reports on the role of arginine metabolism in *C*. *albicans* biofilm formation [42]. In conclusion, this PM-based growth analysis further establishes the role of the Zcf15 and Zcf26 transcription factors in controlling metabolic remodeling during *C*. *albicans* biofilm development. Our genome-wide studies suggested the involvement of Zcf15 and Zcf26 regulators in controlling central metabolism where genes of the glycolysis are down-regulated, whereas genes of the glyoxylate and the TCA cycles are up-regulated. These results also showed an increased expression of genes expressed in the aerobic niche over the fermentative environment. Therefore, we used the triphenyltetrazoliumchloride (TTC) dye reduction test to assess the aerobic state of the strains upon overexpression of *ZCF15* and *ZCF26*. TTC generates a red-color stable compound (formazan) strictly in anaerobic conditions [43]. The wild-type, P_*TDH3*_-*ZCF15* and P_*TDH3*_-*ZCF26* strains were grown overnight and spotted on YPD plates. The TTC assay was performed after 24 h of growth. Interestingly, overexpression of *ZCF15* resulted in the production of a lighter color than the wild-type, whereas no change was observed when *ZCF26* was overexpressed (**Fig 5B**). In conclusion, our results suggest that overexpression of *ZCF15* promotes an aerobic niche that inhibits *C*. *albicans* biofilm formation.

### *zcf26* knockout develops a robust and enhanced biofilm

**Fig 6:**
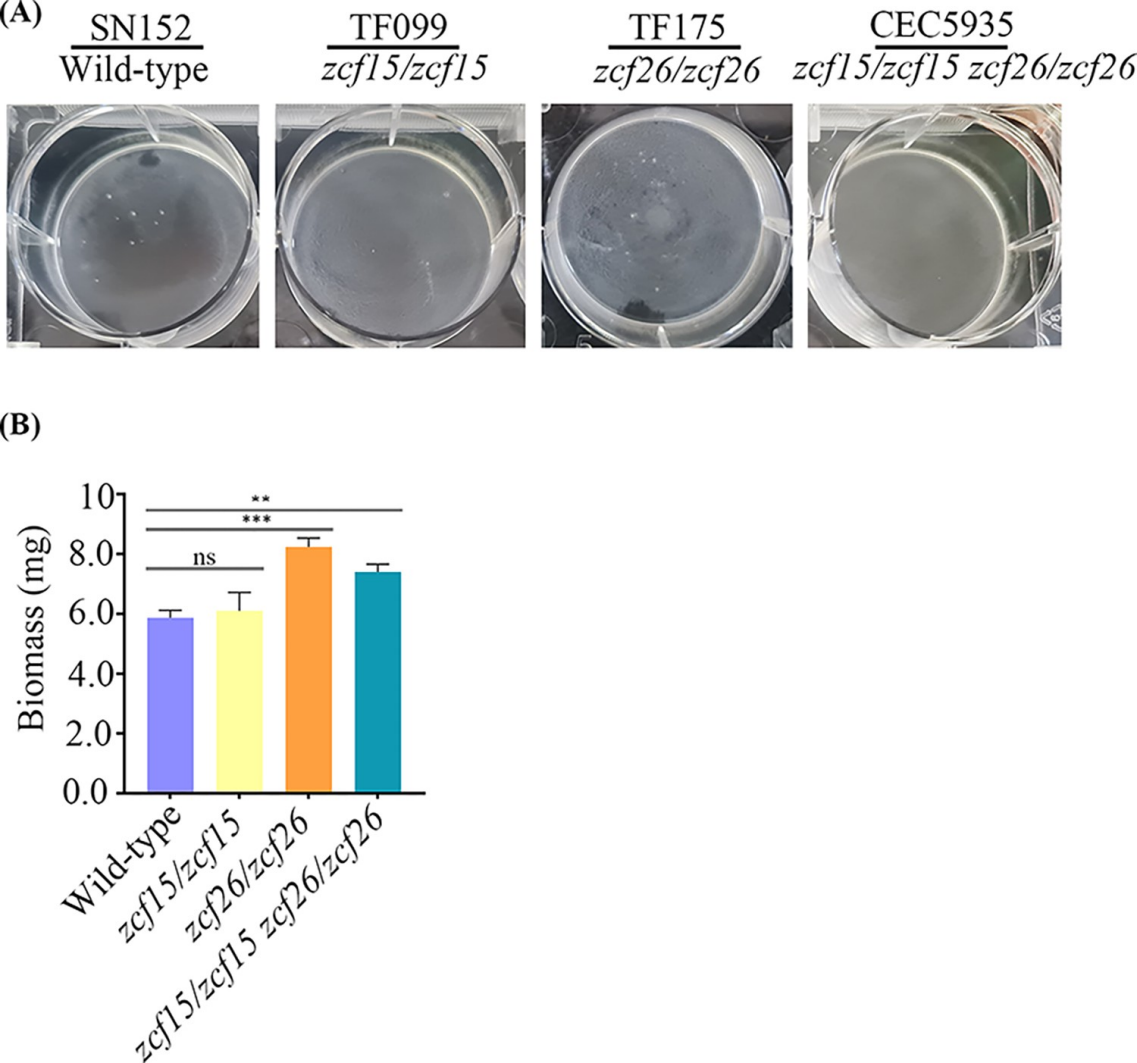
Knockout strain of *zcf26* and double mutant of *zcf15* and *zcf26* develop robust biofilms. Wild-type (SN152) and knockout strains for *zcf15* (TF099), *zcf26* (TF175) or both *zcf15* and *zcf26* (CEC5935) were allowed to adhere to silicone squares in 6-well polystyrene plates in Spider medium at 37°C for 1 h. Biofilms were allowed to grow for 24 h at 110 rpm and photographed. (**B**) Wild-type (SN152) and knockout strain for *zcf15* (TF099), *zcf26* (TF175), or both *zcf15* and *zcf26* (CEC5935) were allowed to adhere to silicone squares in 6-well polystyrene plates in Spider medium at 37°C for 1 h. Biofilms were allowed to grow for 24 h at 110 rpm and dry biomass was measured. Statistical significance was determined using Holm–Sidak method by performing multiple *t* tests; ns: *P* > 0.05; **P ≤ 0.01; ***: *P* ≤ 0.001. The data underlying this figure can be found in S8 Data.

Since overexpression of *ZCF15* and *ZCF26* resulted in reduced biofilm formation, we were curious to evaluate the biofilm formation in the knockout strains of *ZCF15* and *ZCF26*. We could not find enhancement in biofilm formation in the case of the *zcf15* null mutant strain (**Fig 6**). However, the *zcf26* knockout strain formed enhanced biofilms as compared to the wild-type strain (**Fig 6**). Since *ZCF26* overexpression resulted in defect in filamentation, we also examined the filamentation behavior of a *zcf26* knockout strain by spot assay either on solid YPD medium containing 10% FBS or on complete medium. Interestingly, deletion of *zcf26* resulted in an enhanced filamentation on solid surfaces, opposite to the *ZCF26* overexpression phenotype (**S8 Fig**). We also examined the effect of deleting both *zcf15* and *zcf26*. Our results show that a strain carrying a double deletion of *zcf15* and *zcf26* develops a more robust biofilm than wild-type cells (**Fig 6**).

## Discussion

In their natural environment, many microbial species, including bacteria, archaea, and fungi, alternate between planktonic and sessile states, alone or in association with other microbial species [6,44]. A radical shift in gene expression and cellular metabolism has been reported in bacteria and fungi during the transition from planktonic to community growth. Bacterial and fungal biofilms indeed show unique metabolic patterns, such as differential expression of glycolytic pathway and TCA cycle genes, indicating significant metabolic reprogramming during microbial biofilm development [45–47].

Fungal biofilm formation is a complex developmental process that is associated to multiple traits, with each trait having a specific role during the transition from planktonic to biofilm growth. These traits are regulated by a different set of transcription regulators [48,49]. During *C*. *albicans* biofilm establishment, 2 major events occur: cell differentiation and metabolic reprogramming [19,21,42,50,51]. The regulators and their genetic networks modulating cell differentiation during *C*. *albicans* biofilm formation have been extensively studied. For instance, Ace2, Brg1, Efg1, Ndt80, Tec1, Flo8, and Ume6 regulate the expression of genes involved in *C*. *albicans* morphogenesis, which provides architectural stability to biofilms. In contrast, transcription regulators that modulate metabolic alterations during *C*. *albicans* biofilm formation have received less attention [49].

In this study, a large-scale overexpression approach identified a new set of transcription regulators involved in biofilm formation, associated with either morphogenesis (*NRG1*, *RBF1*, *ZFU2*, and *ZCF8*), metabolic alteration (*ZCF15*), or both (*ZCF26*). We selected Zcf15 and Zcf26 for further study as they are paralogs whose occurrence is restricted to CTG clade species that form biofilms, and no prior information on their role in morphogenesis or biofilm formation was known. Moreover, Zcf15 and Zcf26 share a similar binding motif but show differences in their function, perhaps due to differences in protein size and structure at the C-terminus.

Metabolic reprogramming is one of the major changes that occur during microbial biofilm formation [17,19–21]. Bonhomme et al. and colleagues demonstrated the up-regulation of glycolysis genes during *C*. *albicans* biofilm formation and highlighted the role of Tye7 in their regulation [15]. Furthermore, a comparative metabolomic study of *C*. *albicans* planktonic and biofilm cells revealed differential production of metabolites of the TCA cycle, lipid synthesis, amino-acid metabolism, glycolysis, and oxidative stress [21]. These authors showed that the level of citrate decreased in all stages of biofilm formation, including early and intermediate biofilms, while other intermediates of the TCA cycle (succinate, fumarate, and malate) decreased only in mature biofilms. Moreover, comparison of transcript profiling of cells from planktonic cultures and biofilms also highlighted the role of the TCA cycle and mitochondrial activities during *C*. *albicans* biofilm formation [20]. These results suggest an inhibition of the TCA cycle during biofilm maturation and a reduction of the aerobic respiration rate in biofilm cells. Interestingly, transcript profiling of *ZCF15* and *ZCF26* overexpression strains demonstrated their role in the alteration of central metabolism, in particular, the down-regulation of genes of the glycolysis and up-regulation of genes of the glyoxylate pathway and the TCA cycle. In addition to the alterations of genes of the central metabolism, *ZCF26* overexpression also impacted the expression level of genes associated with morphogenesis, which may be the cause of the defect in filamentous growth in the presence of doxycycline. For instance, *ACE2*, *BRG1*, *CPH2*, *EFG1*, *FKH2*, *ASH1*, or *RAS1*, which are involved in the yeast to hyphae transition, are down-regulated when *ZCF26* is overexpressed. On the contrary, most genes whose expression is altered upon *ZCF15* overexpression are associated with metabolism including glyoxylate and the TCA cycles, and no significant differences were observed for genes associated with morphogenesis (**Fig 3A**). These data were well supported by the genome-wide binding study and the locus-specific PCR (**Fig 4A** and **4C**). ChIP-sequencing and ChIP-qPCR experiments revealed that both Zcf15 and Zcf26 bind to the regulatory region of *IDP2*, *MDH1-3*, and *OSM2*, involved in glyoxylate and TCA cycles. Moreover, both regulators bind to the promoters of *TYE7* and *PFK26*, encoding regulators of glycolysis. Interestingly, both overexpressing or deleting *ZCF15* result in a decrease of *TYE7* expression levels, suggesting the involvement of several regulators to control this master regulator of glycolysis. Of note, the deletion of *ZCF26* does not impact *TYE7* expression levels. These results demonstrate their direct role in modulating the expression levels of TCA cycle and glyoxylate cycle genes. In addition, Zcf15 and Zcf26 bind to the promoters of acetyl-CoA synthetase-encoding genes, *ACS1* and *ACS2*, that regulate the metabolism of nonfermentable carbon sources via gluconeogenesis, glyoxylate cycle, and β-oxidation [52]. Phenotypic microarray results further highlighted the involvement of these 2 regulators in controlling metabolic remodeling; indeed, overexpression of *ZCF15* and *ZCF26* resulted in increased growth when precursors of the TCA cycle including succinic acid, α-keto-glutaric acid, and pyruvic acid were used as a carbon source. Based on these results, we posit that upon overexpression of *ZCF15* and *ZCF26*, an alteration in the rate of the glycolysis, TCA cycle, and glyoxylate cycle leads to the establishment of a non-fermentative environment, that favors the planktonic mode of growth and thus results in an impaired biofilm formation.

Besides regulating genes of the central metabolism, Zcf15 and Zcf26 also directly regulate the expression of genes necessary for normal biofilm growth (*CRZ2*, *CSA2*, *RAS1*), involved in biofilm matrix formation (*GCA1*, *GCA2*), and of transcription regulators of biofilm gene networks (*BRG1*, *TEC1*) [6,53–55].

Apart from carbohydrate metabolism, amino-acid metabolism is also crucial for *C*. *albicans* biofilm formation. Garcia-Sanchez and colleagues observed that amino acid biosynthetic pathway genes are up-regulated during biofilm formation under the aforementioned growth conditions, which led to the demonstration of a role in *C*. *albicans* biofilm formation of the *GCN4* gene encoding a master regulator of amino acid biosynthetic genes [19]. In addition, Rajendran and colleagues have shown that amino acid biosynthetic pathway genes such as arginine and proline are up-regulated in high biofilm forming *C*. *albicans* isolates versions [42]. Moreover, a recent study revealed the role of the regulator of amino acid permeases Stp2 in *C*. *albicans* adherence and biofilm maturation [16]: *stp2* knock-out mutants are impaired for amino acid uptake and compensatory mechanisms in nutrient acquisition. We noticed a lower utilization of L-arginine by *ZCF15* and *ZCF26* overexpression strains when used as either carbon source, nitrogen source, or provided as nutritional complement (**Fig 5**). In addition, overexpression of *ZCF15* and *ZCF26* resulted in slower growth when Arg-containing dipeptides were used as a nitrogen source. Strikingly, Zcf26 binds directly to the promoter region of *STP2*, which is also down-regulated during *ZCF26* overexpression. Therefore, this study establishes the role of arginine metabolism during *C*. *albicans* biofilm formation.

Interestingly, the presence of the transcription regulators Zcf15 and Zcf26 is limited to species in the Candida clade that can form biofilms, suggesting their relatively recent acquisition in biofilm-forming species. Furthermore, the shared regulation of several genes by Zcf15 and Zcf26 argues for a common evolutionary origin of these 2 regulators and may allow tight regulation of the set of regulated genes. However, they also have a distinct regulatory networks, and this despite sharing the same binding site, indicative of a probable interaction with coregulators.

In summary, by using overexpression approaches, we discovered new biofilm regulators with either a role in architectural stability and/or a role in metabolic reprogramming. This study also identified several other regulators and genes whose further study will provide a better understanding of the mechanism of *C*. *albicans* biofilm formation. Altogether, this study highlights the role of metabolic reprogramming and its fine-tuned regulation during the shift from planktonic to biofilm growth. This could lead to the development of new antifungals designed to selectively disrupt the fungus central metabolism to treat biofilm-related infections.

## Materials and methods

### Ethics statement

All animal procedures were approved by the Institutional Animal Care and Use Committee at the University of Wisconsin according to the guidelines of the Animal Welfare Act, the Institute of Laboratory Animal Resources Guide for the Care and Use of Laboratory Animals, and Public Health Service Policy under protocol MV1947. Ketamine and xylazine were used for anesthesia. CO_2_ asphyxiation was used for euthanasia at the end of study.

### Media and growth conditions

*C*. *albicans* strains used in this study are listed in **S1 Table**. Cells were grown in YPD (1% yeast extract, 2% peptone, and 2% dextrose) or Spider (1% peptone, 1% yeast extract, 1% mannitol, 0.5% NaCl, and 0.2% K_2_HPO_4_) at 30°C or 37°C for planktonic or biofilm growth, respectively. Solid media were obtained by adding 2% agar. Induction of P_*TET*_ was achieved by adding 25 μg/ml doxycycline. Hyphal growth was induced by adding 10% or 20% FBS to the medium.

### Biofilm measurement by standard optical density assay

To measure the extent of *C*. *albicans* biofilm formation, we performed 96-well standard optical density assays [31] for all *C*. *albicans* doxycycline-dependent P_*TET*_ overexpression strains. Biofilms were allowed to grow at the bottom of 96-well polystyrene plate (pretreated overnight at room temperature with FBS and rinsed with 1× PBS) in YPD medium at 37°C for 18 h at 110 rpm with or without adding 25 μg/ml doxycycline. Optical density was measured using Tecan I control infinite M200. We measured the optical density at 9 independent locations per well; values from 6 independent wells were used to plot the graph and to estimate the statistical significance.

### In vitro biofilm formation and dry biomass measurement

To measure the dry biomass produced, biofilms were grown in 12-well polystyrene TPP plates (Cat. No. 92412) in 2 ml of YPD medium with or without 25 μg/ml doxycycline. The plates were pretreated overnight at room temperature with FBS and rinsed with 1× PBS, then inoculated with cells at OD_600_ = 0.2 and incubated at 37°C for 60 min at 110 rpm agitation for initial adhesion of the cells. After 60 min, the plates were washed with 2 ml of 1× PBS, and 2 ml of fresh YPD medium with or without 25 μg/ml doxycycline were added. Plates were then sealed with breathseal sealing membranes (Greiner bio-one) and incubated at 37°C for 18 h with shaking at 110 rpm. Then, the medium was aspirated, and the wells were gently washed with 1× PBS. To estimate the dry biomass of biofilms produced, biofilms were scrapped, and the content of each well was transferred to pre-weighed nitrocellulose filters. Biofilm-containing filters were dried overnight at 60°C and weighed. The average total biomass for each strain was calculated from 3 independent samples after subtracting the mass of the empty filters [56].

### CLSM for biofilm imaging

Biofilms were grown on silicone squares pretreated overnight at room temperature with FBS and rinsed with 1× PBS. Cells were grown overnight in YPD with 25 μg/ml doxycycline, diluted to OD600 = 0.2 in YPD medium supplemented with 25 μg/ml doxycycline, and allowed to adhere on silicone squares for 1 h at 37°C. Then, the silicone square were rinsed in PBS, fresh medium added, and incubated at 37°C for 18 h. The medium was discarded, and silicone squares were gently washed with 1× PBS and stained with 50 μg/ml of concanavalin A-Alexa Fluor 594 (Invitrogen) at 30°C for 2 h, with gentle shaking at 110 rpm. Silicone squares were then placed in a Petri dish and covered with 1× PBS. Biofilms were imaged as described previously [22]: CSLM was performed at the UtechS PBI facility of Institut Pasteur using an upright LSM700 microscope equipped with a Zeiss 40X/1.0 W plan-Apochromat immersion objective. Images were acquired and assembled into maximum intensity Z-stack projection using the ZEN software.

### RNA extraction and cDNA synthesis

RNAs were isolated using the RNeasy mini kit mirVana RNA isolation kit (Qiagen). Briefly, *C*. *albicans* strains were grown in YPD medium either in planktonic grown at 30°C or biofilm-growth conditions grown at 37°C in shaking mode for 18 h in polystyrene plates. Total RNA was isolated from 4 independent planktonic or biofilm cultures for each strain. Planktonic cells were grown in 50 ml YPD medium in flasks at 30°C till OD600 = 0.8, whereas biofilms were grown in 2 ml of YPD in 12-well polystyrene plates at 37°C for 18 h. Cells were harvested by centrifugation at 4,000 rpm both from planktonic and biofilms isolated cells and washed 3 times with 1× PBS and pelleted at 4,000 rpm. Cells were resuspended in 700 μl of extraction buffer and lysed by adding 0.5 mm of 500 μl of glass beads. Cells were broken in a bead-beater with 500 μl of 0.5 mm of glass beads (6 cycle of 2 min at 10). The RNeasy columns were used to isolate the total RNA. To remove the potential contaminating chromosomal DNA, RNA samples were treated on-column with DNAse for 15 min at room temperature (Cat. No. 79254, Qiagen). A total of 1 μg of purified RNA was used to make cDNA by adding gDNA wipeout (2 μl), RT buffer 5× (4 μl) RT primer mix and Reverse transcriptase (1 μl) (Qiagen, Cat. No. 205311) added in a final volume of 20 μl. Reactions were carried out at 42°C for 15 min followed by heat inactivation at 95°C for 3 min.

### RNA sequencing and analysis

Libraries were built using a TruSeq Stranded mRNA library Preparation Kit (Illumina, United States of America) following the manufacturer’s protocol. Quality control was performed on a BioAnalyzer 2100 (Agilent Technologies), and 75-bp single-end RNA sequencing was performed on the Illumina NextSeq 500 platform.

The RNA-seq analysis was performed with Sequana [57]. In particular, we used RNA-seq pipeline (v0.9.16, https://github.com/sequana/sequana_rnaseq) built on top of Snakemake 5.8.1 [58]. Reads were trimmed from adapters using Cutadapt 2.10 [59], then mapped to the *C*. *albicans* (SC5314, version A22-s07-m01-r105) genome assembly and annotation from Candida Genome Database [60] using STAR 2.7.3a [61]. FeatureCounts 2.0.0 [62] was used to produce the count matrix, assigning reads to features with strand-specificity information. Quality control statistics were summarized using MultiQC 1.8 [63]. Statistical analysis on the count matrix was performed to identify differentially regulated genes, comparing biofilm and planktonic condition RNA expression. Clustering of transcriptomic profiles were assessed using a principal component analysis (PCA). Differential expression testing was conducted using DESeq2 library 1.24.0 [64] scripts based on SARTools 1.7.0 [65] indicating the significance (Benjamini–Hochberg adjusted *p*-values, false discovery rate (FDR) < 0.05) and the effect size (fold-change) for each comparison. Functional categorization of up- and down-regulated genes were achieved by using FungiFun2 [38].

### Quantitative PCR

*C*. *albicans* wild-type (CEC4665) and P_*TET*_*-ZCF15* and P_*TET*_*-ZCF26* strains were grown in biofilm forming condition in the presence of doxycycline as described earlier. RNAs were isolated as described above in RNA extraction section (Qiagen). The integrity of RNAs were examined on 1% agarose gel. cDNA was synthesized by reverse transcription using QuantiTech Reverse Transcription Kit. Primers designed for real time PCR reactions are listed in **S2 Table**. Analysis of melting curves were performed to ensure specific amplification without any secondary nonspecific amplicons (melting curve temperatures used were 80°C (*TEF3*), 77°C (*ECE1*), 83°C (*HWP1*), 80°C (*HSP104*), 78°C (*HYR1*), 83°C (*ZCF15*), 80°C (*ZCF26*), 80°C (*INO1*), 80.5°C (*ORF19*.*4571*), and 81.5°C (*IHD1*)). PCR was carried out in a final volume of 20 μl using SsoAdvanced Universal SYBR Green supermix (BIO-RAD). The real time PCR analysis was achieved with an i-Cycler (BIO-RAD) using the following reaction conditions: 95°C for 2 min, then 40 cycles of 95°C for 30 s, 55°C for 30 s, 72°C for 30 s. Fold difference in expression of mRNA was calculated by the ΔΔC_T_ method (real–time PCR applications guide BIO-RAD) [66] using *C*. *albicans* transcription elongation factor 3 (*TEF3*) transcript as normalization control.

### In vivo rat catheter biofilm formation

To perform in vivo biofilms, the rat central-venous catheter infection model was used, as described previously [13,37,67,68]. To achieve the in vivo *C*. *albicans* biofilm formation, specific pathogen free Sprague Dawley rats weighing 400 g each were used. A heparinised (100 U/ml) polyethylene catheter with 0.76 mm inner and 1.52 mm outer diameters was inserted into the external jugular vein. The catheter was secured to the vein with the proximal end tunneled subcutaneously to the midscapular space and externalized through the skin. The catheters were inserted 24 h prior to infection to permit a conditioning period for a deposition of host protein on the catheter surface. Infection was achieved by intraluminal instillation of 500 μl *C*. *albicans* cells (10^6^ cells/ml). After a 4 h dwelling period, the catheter volume was withdrawn, and the catheter flushed with heparinized 0.15 M NaCl. Catheters were removed after 24 h of *C*. *albicans* infection to assay biofilm development on the intraluminal surface by SEM. Catheter segments were washed with 0.1 M phosphate buffer, pH 7.2, fixed in 1% glutaraldehyde/4% formaldehyde, washed again with phosphate buffer for 5 min, and placed in 1% osmium tetroxide for 30 min. The samples were dehydrated in a series of 10 min ethanol washes (30%, 50%, 85%, 95%, and 100%), followed by critical point drying. Specimens were mounted on aluminum stubs, sputter coated with gold, and imaged using a Hitachi S-5700 or JEOL JSM-6100 SEM in the high-vacuum mode at 10 kV. Images were processed using Adobe photoshop software.

### Chromatin immunoprecipitation (ChIP)

The ChIP assays were performed as described previously [69]. Briefly, each strain was grown in biofilm condition for 18 h and cells were cross-linked with 1% final concentration of formaldehyde for 25 min at 30°C. Chromatin was isolated and sonicated to yield an average fragment size of 300 to 500 bp. The DNA in 50 μl of water was immunoprecipitated with 20 μg/ml anti-protein A antibodies (Sigma Aldrich) and purified by phenol/chloroform extraction. The total, immunoprecipitated (IP) DNA, and beads only material were used to determine the binding of Zcf15 and Zcf26 across the genome by ChIP-sequencing, or to the promoters of a subset of biofilm-related genes by real time PCR (qPCR), as described before. The template used was as follows—1 μl of a 1:50 dilution for input and 1 μl of a 1:3 dilution for immunoprecipitated DNA (IP) Zcf15-TAP, Zcf26-TAP, and an untagged control strain. The conditions used for qPCR were as follows: 95°C for 2 min, then 40 cycles of 95°C for 30 s, 55°C for 30 s, 72°C for 45 s. The results were analyzed using CFX Manager Software. The graph was plotted according to the percent input method [70].

### Library preparation and ChIP-sequencing analysis and DNA binding motif identification

The ChIP DNA library was prepared using TruSeq ChIP sample preparation guidelines (Illumine) and sequencing was achieved by using Nextseq 500 run. The ChIP-seq analysis was performed with the ChIP-seq pipeline of the Sequana framework [57]. We checked the quality of the data by computing the ratio between data peak and so-called phantom peaks and found values >1.3, which indicates a good-quality ChIP-seq data according to best practices recommended by ENCODE [71]. We then mapped the data and identify narrow and broad peaks using Macs3 (https://github.com/macs3-project/MACS). Finally, we obtained the final list of peaks by computing IDR (irreproducible discovery rate), which is the approach used in ChIP-sequencing analysis to provide stable thresholds based on reproducibility [72]. The DNA binding motif across the *C*. *albicans* genome was identified using Motif Analysis of Large Nucleotide Datasets (MEME-ChIP) [39]. The interaction network was generated with Cytoscape [73].

### Phenotype microArray and data analysis

Phenotypic microarray **(**PM) plates and reagents (inoculating fluid IFY-0 base, redox dye mix D and E) were purchased from Biolog Inc. The composition of the PM plates can be found on the Biolog website (https://www.biolog.com/wp-content/uploads/2020/04/00A-042-Rev-C-Phenotype-MicroArrays-1-10-Plate-Maps.pdf). *C*. *albicans* strains were streaked to YPD plates and grown for 2 days at 30°C. A total of 2 to 6 colonies from each YPD plates were transferred to 15 ml tubes in NS medium (nutrient supplement) and cell density was calculated using turbidimeter (Biolog). Turbidity of the suspension was measured by turbidimeter (Biolog) and transmittance was reached to 62%T (+/−1%). The PM panels represent 96-well plates containing different substrate in each well. In addition to the different substrate, PM wells were also containing the minimal components required for normal growth and prepared according to the manufacturer’s guidelines. PM additives and dye were added according to the method provided by Biolog Inc. In summary, 0.5 ml of cell suspension were mixed to appropriate volume of PM inoculating fluids, and a 100 μl of different cell suspension from the PM inoculating fluid was transferred to each well coated with different nutrients. Plates were sealed with PCR seal to keep wells from drying out and to avoid cross-well spreading of volatile chemicals. All PM plates were incubated in Omnilog at 30°C for 96 h. The Omnilog software was used to analyze the data. Differential growth was considered when area under the curve (AUC) of mutants were differed by 2 times in both directions as compared to the reference strain. Differential growth was converted in the form of a heat-map using Heatmapper [74]. Clustering was achieved by average linkage and distance was measured by using Pearson method.

### TTC assay

To analyze the red color pigmentation of TCC, *C*. *albicans* strains were spotted on YPD plates and incubated 24 h at 30°C. Then, approximately 10 ml of 0.025% TTC in 1% agarose solution were poured on top of the plates, incubated for 30 min at room temperature, and photographed using a Phenobooth (Singer Instruments).

### Statistical significance

Graphs were generated using GraphPad Prism. Statistical significance was determined by performing multiple *t* test using Holm–Sidak method [75].

## Supporting information

S1 Fig Biofilm formation and growth measurements for candidate genes identified by overexpression approach. **(A)** *C*. *albicans* wild-type and P_*TET*_-overexpression strains were grown overnight in YPD medium with or without 25 μg/ml doxycycline. Biofilm formation was allowed to develop in 96-well polystyrene plates in YPD medium with or without 25 μg/ml doxycycline at 37°C for 18 h. (**B**) Wild-type (CEC4665) and P_*TET*_*-*overexpression strains were grown in liquid YPD medium, with or without 25 μg/ml doxycycline until the stationary phase was reached. Optical density was measured using Tecan Sunrise.ns: *P* > 0.05; **P ≤ 0.01. The data underlying this figure can be found in S9 Data.
(PDF)

S2 Fig Transcription factors *ZCF15* and *ZCF26* are paralogous genes. (**A**) The extent of filamentation of wild-type (CEC4665), P_*TET*_*-NRG1* (CEC6039), P_*TET*_*-RBF1* (CEC6043), P_*TET*_*-ZCF8* (CEC6053), P_*TET*_*-ZCF15* (CEC6052), P_*TET*_*-ZCF26* (CEC6051), and P_*TET*_*-ZFU2* (CEC6044) strains were examined at the single colony level on YPD plates containing 20% fetal bovine serum with or without 25 μg/ml doxycycline and grown for 5 days at 37°C. (**B**) Similarly, filamentation assay was performed for 1 h in YPD liquid medium with 10% FBS for the indicated strains in the absence or presence of 25 μg/ml doxycycline at 37°C. Scale bars: 20 μm. (**C**) Orthologs of the indicated transcription factors in the budding yeasts of the Saccharomycetes class are shown. The presence (blue box) or absence (empty box) of the orthologs of the transcription factor indicated for each species was shown. This tree is illustrative as the branches are not drawn to the scale. Non-candida species are: *Meyerozyma guilliermondii*, *Yamadazyma tenuis*, *Debaryomyces hansenii*, *Suhomyces tanzawaensis*, *Scheffersomyces stipitis*, *Spathaspora passalidarum*, *Lodderomyces elongisporus*, *Naumovozyma castellii*, *Saccharomyces kudriavzevii*, and *Saccharomyces cerevisiae*. (**D**) Phylogenetic analyses show that *ZCF15*, *ZCF25* and *ZCF26* transcription factors are paralogous genes. Non-candida species are as follows: *Lodderomyces elongisporus*, *Spathaspora passalidarum*, *Scheffersomyces stipitis*, and *Debaryomyces hansenii*. The evolutionary history was inferred using the Neighbor-Joining method. The percentages of replicate trees in which the taxa clustered together in the bootstrap test (1,000 replicates) are shown next to the branches. Evolutionary analyses were generated using the MEGA X software. The data underlying this figure can be found in S10 Data.
(PDF)

S3 Fig *ZCF25* overexpression phenotype is not similar to *ZCF15* and *ZCF26*. **(A)** AlphaFold structure of Zcf15 and Zcf26 proteins were compared using Molstar viewer. (**B**) Biofilm formation assay of strains with overexpression strains of *ZCF25* (CEC5932) were allowed to form biofilms in 12-well polystyrene microtiter plates in YPD medium at 37°C for 18 h before and dry weight biomass was estimated. (**C**) The extent of filamentation of the strains was estimated by spot assay on YPD plates containing 20% fetal bovine serum. The data underlying this figure can be found in S11 Data.
(PDF)

S4 Fig Constitutive expression of transcription factors *ZCF15* and *ZCF26* results in reduced biofilm formation. **(A)** Biofilm assay was performed with wild-type (CEC4665), P_*TET*_*-ZCF15* (CEC6052), P_*TET*_*-ZCF26* (CEC6051) strains in Spider medium with or without 25 μg/ml doxycycline and grown for 18 h at 37°C. (**B**) Biofilm formation assay of strains with constitutive expression of *ZCF15* or *ZCF26* placed under the control of P_*TDH3*_, a constitutive promoter. The wild-type parental strain (CEC4665), 2 independent strains with P_*TDH3*_*-ZCF15* (CEC5915 and CEC5916) or with P_*TDH3*_*-ZCF26* (CEC5917 and CEC5918) were allowed to form biofilms in 12-well polystyrene microtiter plates in YPD medium at 37°C for 18 h before and dry weight biomass was estimated. (**C**) The extent of filamentation of these strains was estimated by spot assay on YPD plates containing 20% fetal bovine serum. *: *P* ≤ 0.05; **: *P* ≤ 0.01. The data underlying this figure can be found in S12 Data (A) and S13 Data (B).
(PDF)

S5 Fig Comparative analysis of transcriptome profile of P_*TET*_*-ZCF15* and P_*TET*_*-ZCF26*. (**A**) Genome-wide expression data were compared for commonly up-regulated genes between P_*TET*_*-ZCF15* and P_*TET*_*-ZCF26* overexpression strains (blue and yellow circles, respectively) and represented as Venn diagrams. (**B**) Similarly, a Venn diagram was constructed for down-regulated genes in the 2 strains. A total of 221 up-regulated and 410 down-regulated genes are common between the 2 datasets. (**C**) Heat-map illustrating the differentially expressed genes of the glycolytic and tricarboxylic acid pathways when *ZCF15* and *ZCF26* were grown with 25 μg/ml doxycycline in biofilm-forming condition. The data underlying this figure can be found in S14 Data.
(PDF)

S6 Fig TAP-tagged *ZCF15* and *ZCF26* are functional upon overexpression. (**A**) To examine the functionality of TAP-epitope tagged protein, the wild-type parental strain, *N-TAP-ZCF15* (CEC5929) and *N-TAP-ZCF26* (CEC5931) strains were allowed to form biofilms in 12-well polystyrene plates in YPD medium, with or without 25 μg/ml doxycycline at 37°C for 18 h and dry weight biomass measured. (**B**) The extent of filamentation of these strains was estimated by spot assay on YPD plates containing 20% fetal bovine serum with or without 25 μg/ml doxycycline. ns: *P* > 0.05, **: *P* ≤ 0.01, ***: *P* ≤ 0.001. The data underlying this figure can be found in S15 Data.
(PDF)

S7 Fig Metabolic activities of *ZCF15* and *ZCF26* overexpression strains. Comparison of metabolic activity profiles of the parental strain (CEC4665) and the overexpression strain for *ZCF15* and *ZCF26* on indicated PM plates is shown as a heat-map. The data underlying this figure can be found in S16 Data.
(PDF)

S8 Fig *zcf26* deletion resulted in enhanced colony wrinkling. The extent of filamentation of wild-type and *zcf26* null mutant strains was estimated by spot assay on YPD plates containing 10% fetal bovine serum or growing on complete medium at 37°C.
(PDF)

S1 Text Supplementary experimental procedures. (PDF)

S1 Table List of strains used in this study. (PDF)

S2 Table List of oligonucleotides used in this study. (PDF)

S1 Data Transcriptome profile of wild-type and overexpression mutants for *ZCF15* and *ZCF26*, and DNA-binding sites for Zcf15 and Zcf26 determined by ChIP-sequencing. S1A Table contains the transcriptome data obtained on 4 independent samples of the wild-type strains (CEC4665) after doxycycline treatment. S1B and S1C Table contain lists of up- and down-regulated genes during WT strain doxycycline treatment, respectively. S1D and S1E Table contain the complete transcriptome data for P_*TET*_*-ZCF15* doxycycline-treated and P_*TET*_*-ZCF26* doxycycline-treated strains, respectively. S1F and S1G Table contain lists of up- and down-regulated genes during P_*TET*_*-ZCF15* doxycycline-treatment, respectively. S1H and S1I Table contain lists of up- and down-regulated genes during P_*TET*_*-ZCF26* doxycycline-treatment, respectively. S1J and S1K Table contain ChIP-sequencing data for TAP-Zcf15 and TAP-Zcf26, respectively, and S1L Table lists genes bound and with altered expression levels upon TAP-Zcf15 and TAP-Zcf26 overexpression.
(XLSX)

S2 Data Data underlying Fig 1C. (XLSX)

S3 Data Data underlying Fig 1D. (XLSX)

S4 Data Data underlying Fig 1E. (XLSX)

S5 Data Data underlying Fig 3D. (XLSX)

S6 Data Data underlying Fig 4C. (XLSX)

S7 Data Data underlying Fig 5A. (XLSX)

S8 Data Data underlying Fig 6B. (XLSX)

S9 Data Data underlying S1B Fig. (XLSX)

S10 Data Data underlying S2D Fig. (XLSX)

S11 Data Data underlying S3B Fig. (XLSX)

S12 Data Data underlying S4A Fig. (XLSX)

S13 Data Data underlying S4B Fig. (XLSX)

S14 Data Data underlying S5C Fig. (XLSX)

S15 Data Data underlying S6A Fig. (XLSX)

S16 Data Data underlying S7 Fig. (XLSX)

## Abbreviations

AUC: area under the curve
ChIP: chromatin immunoprecipitation
CLSM: confocal laser scanning microscopy
FBS: fetal bovine serum
FDR: false discovery rate
IDR: irreproducible discovery rate
PCA: principal component analysis
PM: phenotypic microarray
RBH: reciprocal best hits
SEM: scanning electron microscopy
TAP: tandem affinity purification
TCA: tricarboxylic acid
TEF3: transcription elongation factor 3
TTC: triphenyltetrazoliumchloride
