# Supplementary figures and images for: Metabolic reprogramming during Candida albicans planktonic-biofilm transition is modulated by the transcription factors Zcf15 and Zcf26
Source: PLoS Biol. 2024 Jun 21;22(6):e3002693. doi: 10.1371/journal.pbio.3002693 (PMC11221756; doi:10.1371/journal.pbio.3002693)

S1 Fig

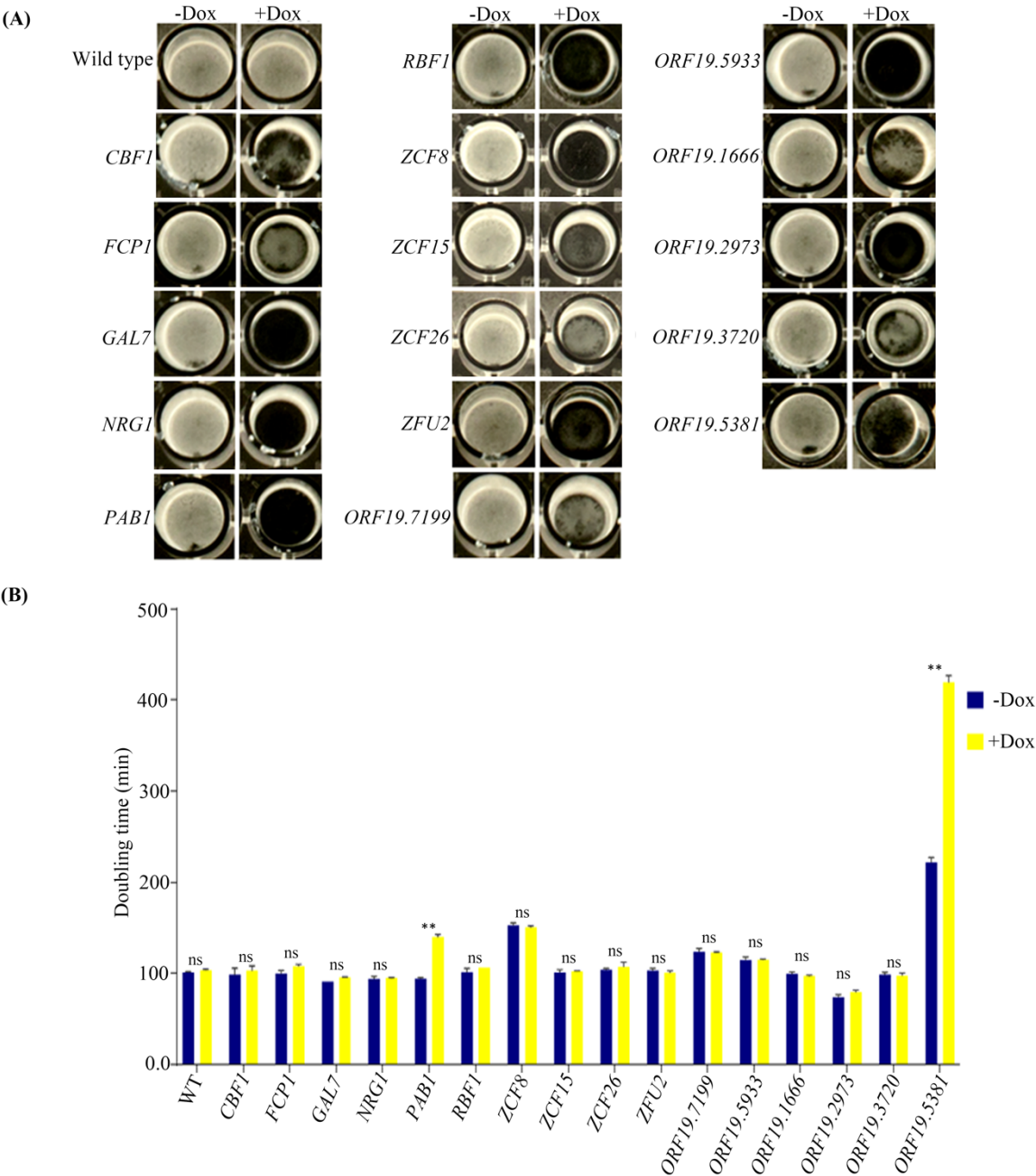

Supplement: S1 Fig — (A) C. albicans wild-type and PTET-overexpression strains were grown overnight in YPD medium with or without 25 μg/ml doxycycline. Biofilm formation was allowed to develop in 96-well polystyrene plates in YPD medium with or without 25 μg/ml doxycycline at 37°C for 18 h. (B) Wild-type (CEC4665) and PTET-overexpression strains were grown in liquid YPD medium, with or without 25 μg/ml doxycycline until the stationary phase was reached. Optical density was measured using Tecan Sunrise.ns: P > 0.05; **P ≤ 0.01. The data underlying this figure can be found in S9 Data. (PDF) [file pbio.3002693.s001.pdf]

S2 Fig

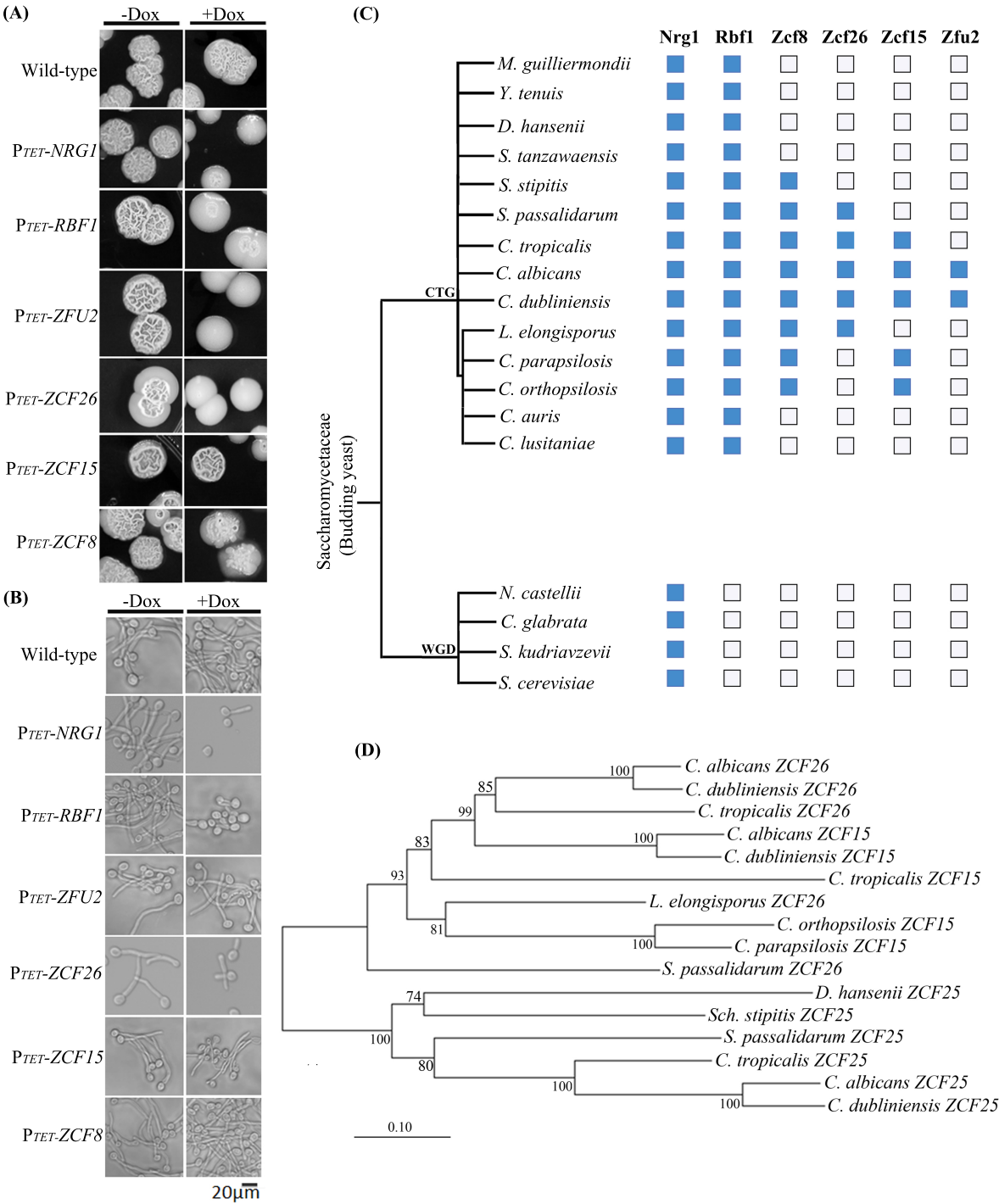

Supplement: S2 Fig — (A) The extent of filamentation of wild-type (CEC4665), PTET-NRG1 (CEC6039), PTET-RBF1 (CEC6043), PTET-ZCF8 (CEC6053), PTET-ZCF15 (CEC6052), PTET-ZCF26 (CEC6051), and PTET-ZFU2 (CEC6044) strains were examined at the single colony level on YPD plates containing 20% fetal bovine serum with or without 25 μg/ml doxycycline and grown for 5 days at 37°C. (B) Similarly, filamentation assay was performed for 1 h in YPD liquid medium with 10% FBS for the indicated strains in the absence or presence of 25 μg/ml doxycycline at 37°C. Scale bars: 20 μm. (C) Orthologs of the indicated transcription factors in the budding yeasts of the Saccharomycetes class are shown. The presence (blue box) or absence (empty box) of the orthologs of the transcription factor indicated for each species was shown. This tree is illustrative as the branches are not drawn to the scale. Non-candida species are: Meyerozyma guilliermondii, Yamadazyma tenuis, Debaryomyces hansenii, Suhomyces tanzawaensis, Scheffersomyces stipitis, Spathaspora passalidarum, Lodderomyces elongisporus, Naumovozyma castellii, Saccharomyces kudriavzevii, and Saccharomyces cerevisiae. (D) Phylogenetic analyses show that ZCF15, ZCF25 and ZCF26 transcription factors are paralogous genes. Non-candida species are as follows: Lodderomyces elongisporus, Spathaspora passalidarum, Scheffersomyces stipitis, and Debaryomyces hansenii. The evolutionary history was inferred using the Neighbor-Joining method. The percentages of replicate trees in which the taxa clustered together in the bootstrap test (1,000 replicates) are shown next to the branches. Evolutionary analyses were generated using the MEGA X software. The data underlying this figure can be found in S10 Data. (PDF) [file pbio.3002693.s002.pdf]

S3 Fig

(A)

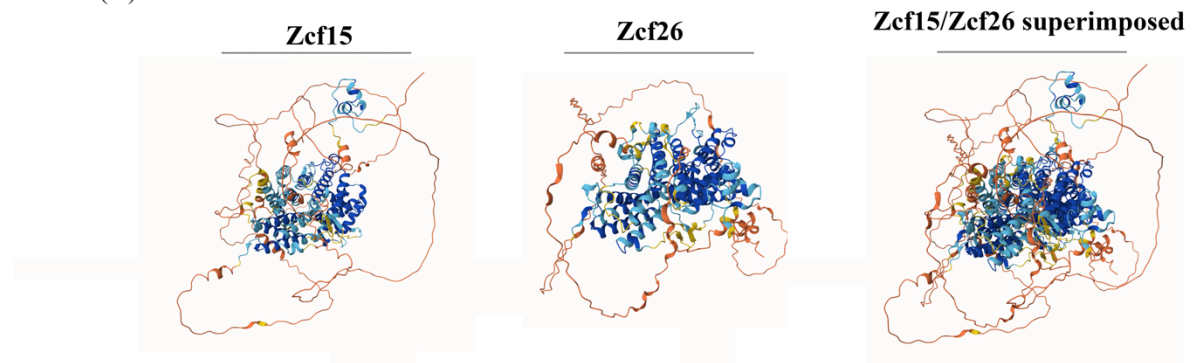

(B)

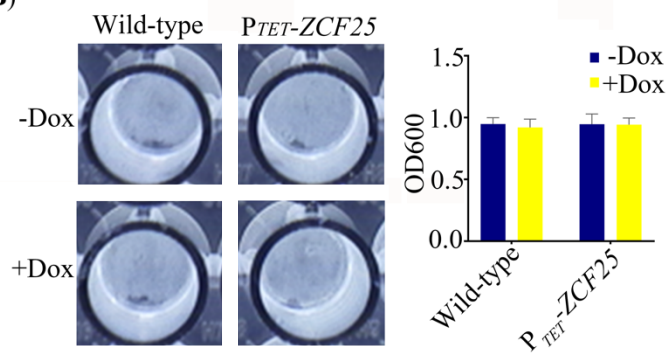

(C)

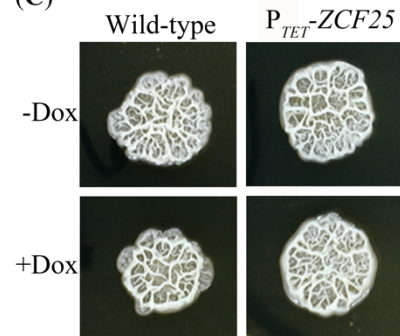

Supplement: S3 Fig — (A) AlphaFold structure of Zcf15 and Zcf26 proteins were compared using Molstar viewer. (B) Biofilm formation assay of strains with overexpression strains of ZCF25 (CEC5932) were allowed to form biofilms in 12-well polystyrene microtiter plates in YPD medium at 37°C for 18 h before and dry weight biomass was estimated. (C) The extent of filamentation of the strains was estimated by spot assay on YPD plates containing 20% fetal bovine serum. The data underlying this figure can be found in S11 Data. (PDF) [file pbio.3002693.s003.pdf]

S4 Fig  
(A)

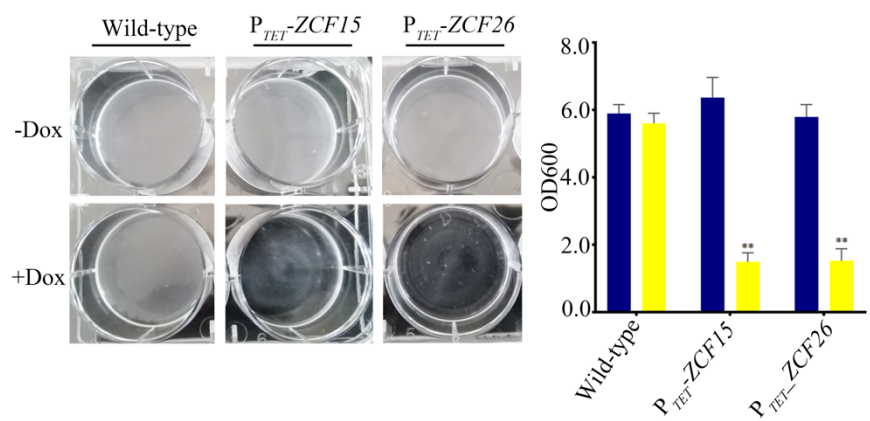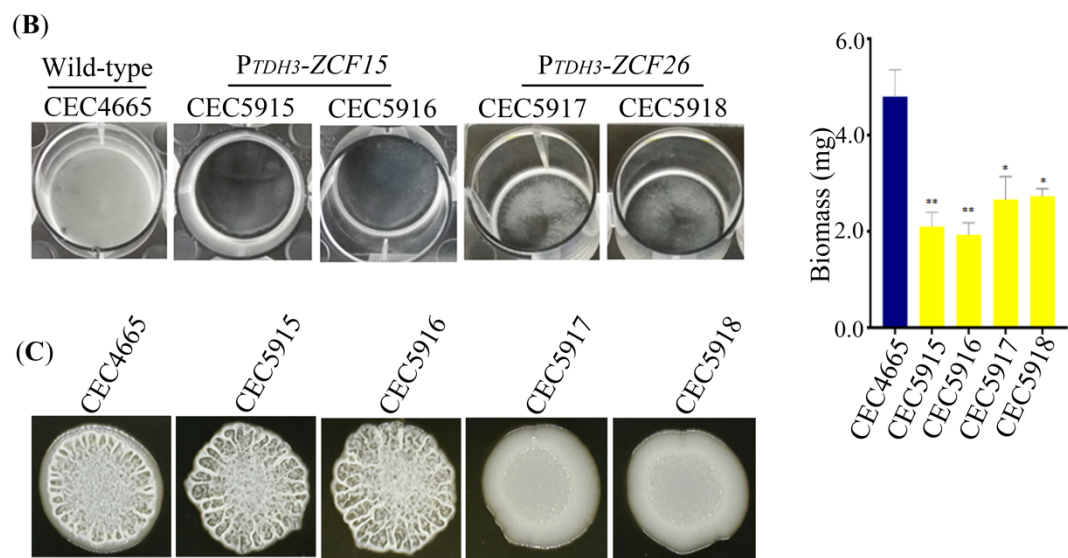

Supplement: S4 Fig — (A) Biofilm assay was performed with wild-type (CEC4665), PTET-ZCF15 (CEC6052), PTET-ZCF26 (CEC6051) strains in Spider medium with or without 25 μg/ml doxycycline and grown for 18 h at 37°C. (B) Biofilm formation assay of strains with constitutive expression of ZCF15 or ZCF26 placed under the control of PTDH3, a constitutive promoter. The wild-type parental strain (CEC4665), 2 independent strains with PTDH3-ZCF15 (CEC5915 and CEC5916) or with PTDH3-ZCF26 (CEC5917 and CEC5918) were allowed to form biofilms in 12-well polystyrene microtiter plates in YPD medium at 37°C for 18 h before and dry weight biomass was estimated. (C) The extent of filamentation of these strains was estimated by spot assay on YPD plates containing 20% fetal bovine serum. *: P ≤ 0.05; **: P ≤ 0.01. The data underlying this figure can be found in S12 Data (A) and S13 Data (B). (PDF) [file pbio.3002693.s004.pdf]

S5 Fig

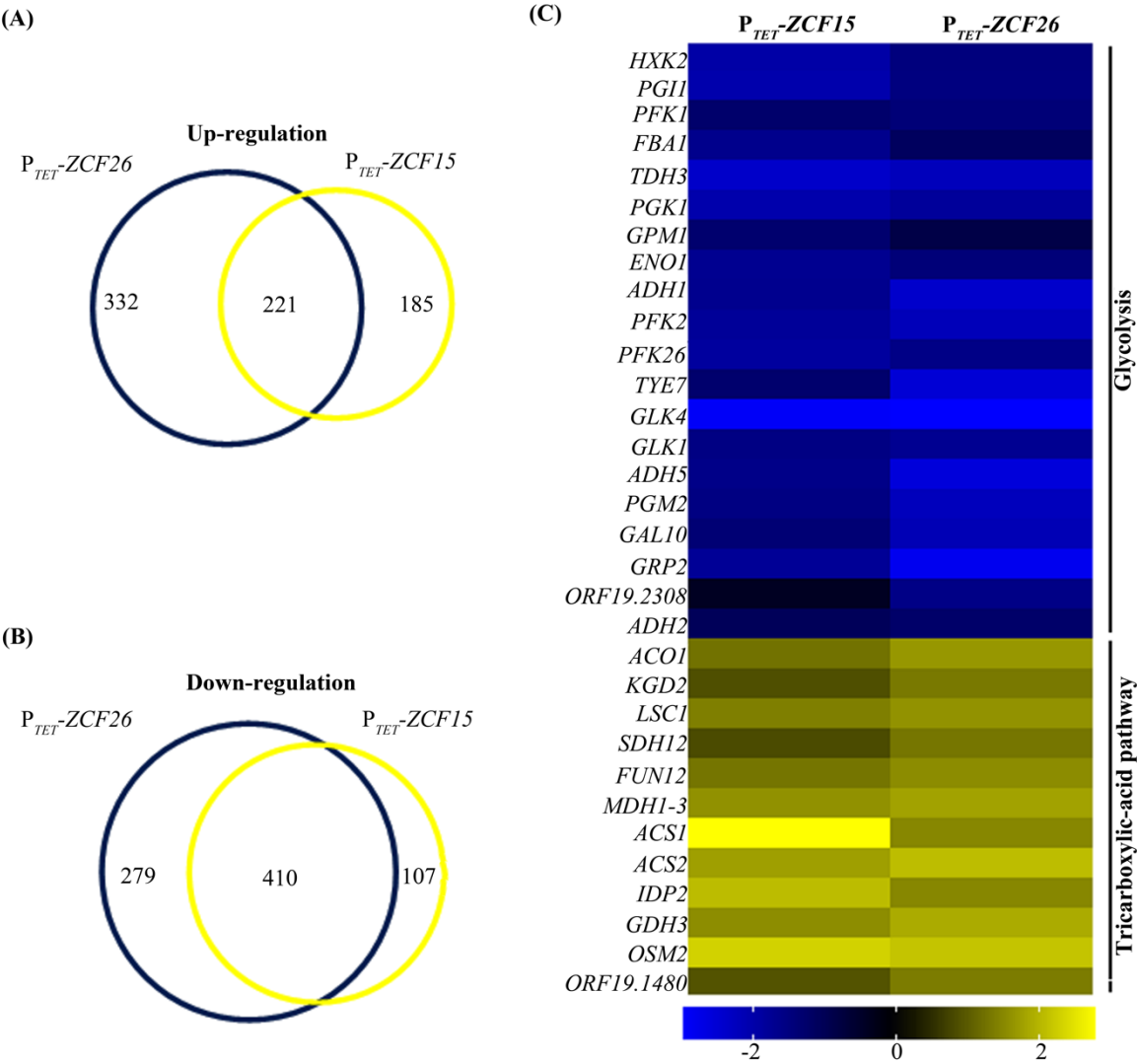

Supplement: S5 Fig — (A) Genome-wide expression data were compared for commonly up-regulated genes between PTET-ZCF15 and PTET-ZCF26 overexpression strains (blue and yellow circles, respectively) and represented as Venn diagrams. (B) Similarly, a Venn diagram was constructed for down-regulated genes in the 2 strains. A total of 221 up-regulated and 410 down-regulated genes are common between the 2 datasets. (C) Heat-map illustrating the differentially expressed genes of the glycolytic and tricarboxylic acid pathways when ZCF15 and ZCF26 were grown with 25 μg/ml doxycycline in biofilm-forming condition. The data underlying this figure can be found in S14 Data. (PDF) [file pbio.3002693.s005.pdf]

S6 Fig

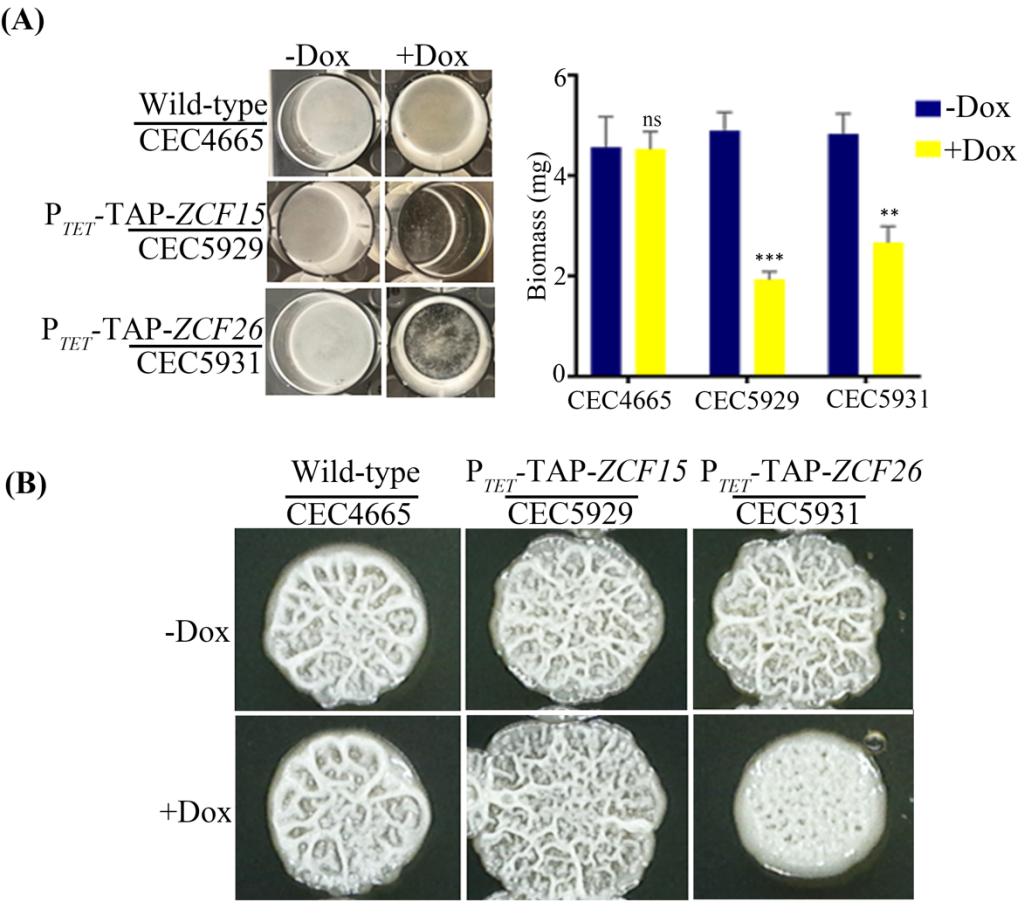

Supplement: S6 Fig — (A) To examine the functionality of TAP-epitope tagged protein, the wild-type parental strain, N-TAP-ZCF15 (CEC5929) and N-TAP-ZCF26 (CEC5931) strains were allowed to form biofilms in 12-well polystyrene plates in YPD medium, with or without 25 μg/ml doxycycline at 37°C for 18 h and dry weight biomass measured. (B) The extent of filamentation of these strains was estimated by spot assay on YPD plates containing 20% fetal bovine serum with or without 25 μg/ml doxycycline. ns: P > 0.05, **: P ≤ 0.01, ***: P ≤ 0.001. The data underlying this figure can be found in S15 Data. (PDF) [file pbio.3002693.s006.pdf]

S7 Fig

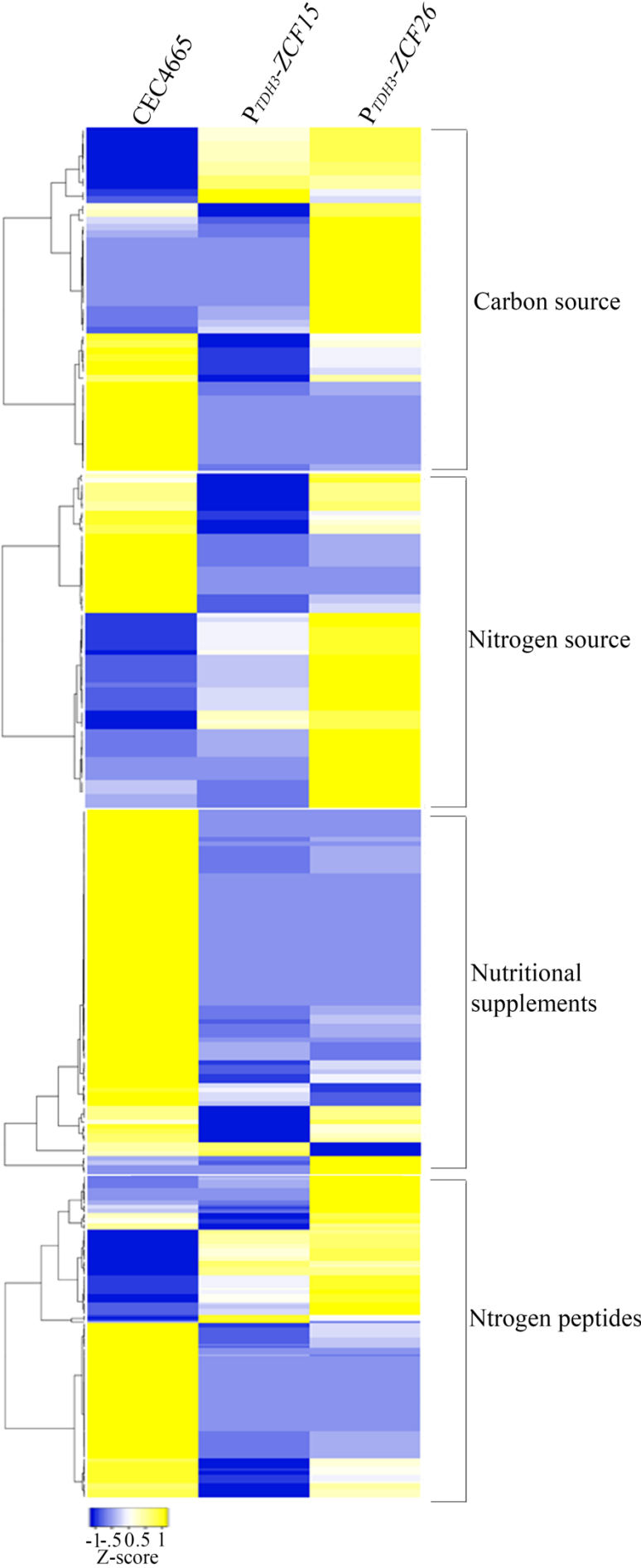

Supplement: S7 Fig — Comparison of metabolic activity profiles of the parental strain (CEC4665) and the overexpression strain for ZCF15 and ZCF26 on indicated PM plates is shown as a heat-map. The data underlying this figure can be found in S16 Data. (PDF) [file pbio.3002693.s007.pdf]

S8 Fig

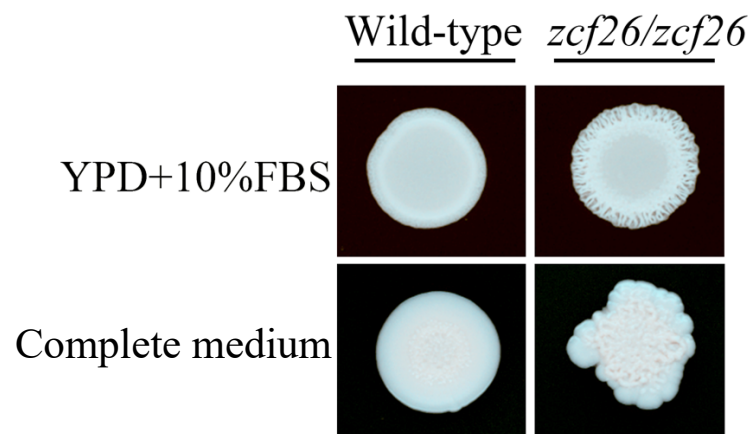

Supplement: S8 Fig — The extent of filamentation of wild-type and zcf26 null mutant strains was estimated by spot assay on YPD plates containing 10% fetal bovine serum or growing on complete medium at 37°C. (PDF) [file pbio.3002693.s008.pdf]
