# Supplementary material for: Metabolic reprogramming during Candida albicans planktonic-biofilm transition is modulated by the transcription factors Zcf15 and Zcf26
Source: PLoS Biol. 2024 Jun 21;22(6):e3002693. doi: 10.1371/journal.pbio.3002693 (PMC11221756; doi:10.1371/journal.pbio.3002693)
Supplement: S1 Text — (PDF) [file pbio.3002693.s009.pdf]

Supporting Experimental Procedures

Orthologues and phylogenetic analysis: Orthologue searches were performed using Hidden Markov Model HMMER Web server [1]. Transcription factors orthologue was also confirmed by Best Reciprocal Hits (RBH). A phylogenetic tree was inferred using the Neighbor-Joining method. The percentage of replicate trees in which the taxa clustered together was determined by performing the bootstrap test (1000 replicates). The evolutionary distances were computed by the Poisson correction method. Evolutionary analyses were conducted using MEGA X[2].

Construction of *C. albicans* overexpression strains: Detailed methods of transferring *C. albicans* ORFs from pDONR207 into the expression plasmids and integrating the resulting expression plasmids at the *RPS1* locus have been described [3,4]. Briefly, ORFs were transferred from the entry clones into our collection of 2496 barcoded P_{TET}-driven overexpression plasmids using the Gateway LR clonase II Enzyme mix (Invitrogen). After *E. coli* DH5α transformation, the ORF transfer into the expression plasmid was verified by *Eco*RV digestion. The *URA3*-bearing expression plasmids were digested by *Stu*I and transformed into *C. albicans* strain CEC4642 (a SN76 derivative, [5]) according to [6], and adapted to the use of 96-well microplates. Transformants were selected for prototrophy and verified by PCR using primer pair CIpUL/CIpUR, which yields a 1 kb amplicon if the integration of the overexpression plasmid has occurred at the *RPS1* locus [7].

Construction of CEC5929, CEC5930, CEC5931 and CEC5932: To examine the genome-wide binding events of *ZCF15* and *ZCF26*, we constructed N-terminal TAP-tagged strains for both *ZCF15* and *ZCF26*. To generate the TAP-tagged strain of *ZCF15* and *ZCF26*, plasmids were isolated from *E. coli* strain ECC1983 and ECC1984 (see below plasmid construction) respectively. *Stu*I-digested DNA fragments were transformed to *C. albicans* strain CEC4642. Transformants were selected for prototrophy and proper integration at the *RPS1* locus was verified by PCR using primer pair CIpUL/CIpUR.

Construction of CEC5915, CEC5916, CEC5917 and CEC5918: Plasmids carrying *ZCF15* (ECC1985) or *ZCF26* (ECC1986) (see below: plasmid construction) under the control of P_{TDH3} were digested by *Stu*I prior to transformation into *C. albicans* strain CEC4642. Transformants were selected for prototrophy and proper integration at the *RPS1* locus was verified by PCR using primers CIpUL and CIpUR.

Construction of CEC5935: A plasmid containing the SAT-flipper cassette flanked by *ZCF15* upstram and downstream regions (ECC1979) was digested by *SacI* and *KpnI* and integrated to the

ZCF15 locus of TF135 [8]. Transformants were selected on YPD containing 152µg/mL nourseothricin, checked by PCR using primers ZCF15USSATF and ZCF15DSSATR. A heterozygous clone was grown overnight at 30°C in YP+2% maltose, and a 1/1000 dilution was spotted on YPD plates containing 25 µg/mL nourseothricin. Small colonies unable to grow on YPD + 120 µg/mL nourseothricin were checked by PCR to confirm excision of the marker, and submitted to a second round of transformation as decribed previously.

Plasmid construction

ECC1979: a 670 bp fragment upstream *ZCF15* and a 451 bp fragment downstream *ZCF15* were PCR amplified with oligos ZCF15USSATF, ZCF15USSATR and ZCF15DSSATF, ZCF15DSSATR, respectively. The upstream fragment was cloned in pSFS2A digested with *Kpn*I and *Xho*I; the downstream fragment was inserted in the resulting plasmid digested with *Sac*I and *Sac*II.

ECC1983 and ECC1984: The Entry plasmids (BP clone) bearing the *ZCF15* or the *ZCF26* coding sequences, respectively, were used in a Gateway LR reaction together with the CIp-P_{TET}-TAP-GTW-SP vector (ECC1095). Recombination mixes were transformed into *E. coli* Top10. The resulting plasmids were digested with *Eco*RV and *Bam*HI to verify the integration of the appropriate ORF.

ECC1985: The Entry plasmid (BP clone) bearing *ZCF15* coding sequences was used in a Gateway LR reaction together with the CIp-CaP_{TDH3}-GTW (ECC843) vector. The recombination mixes were transformed into *E. coli* Top10. The resulting plasmids were digested with *Eco*RV and *Bam*HI to verify the cloning of the appropriate ORF.

ECC1986: The Entry plasmid (BP clone) bearing *ZCF26* coding sequences was used in a Gateway LR reaction together with the ECC843 (CIp-CaP_{TDH3}-GTW) vector. The recombination mixes were transformed into *E. coli* Top10. Transformants were digested with *Eco*RV and *Bam*HI to verify the cloning of the appropriate ORF.

References

1. Finn RD, Clements J, Arndt W, Miller BL, Wheeler TJ, Schreiber F, et al. HMMER web server: 2015 update. Nucleic Acids Res. 2015;43: W30–W38. doi:10.1093/nar/gkv397
2. Kumar S, Stecher G, Li M, Knyaz C, Tamura K. MEGA X: Molecular Evolutionary Genetics Analysis across Computing Platforms. Mol Biol Evol. 2018;35: 1547–1549. doi:10.1093/molbev/msy096
3. Cabral V, Chauvel M, Firon A, Legrand M, Nesseir A, Bachellier-Bassi S, et al. Modular Gene Over-expression Strategies for *Candida albicans*. 2012. doi:10.1007/978-1-61779-539-8_15
4. Chauvel M, Bachellier-Bassi S, Guérout A-M, Lee KK, Maufrais C, Permal E, et al. High-throughput functional profiling of the human fungal pathogen *Candida albicans* genome. Res Microbiol. 2023;174: 104025. doi:10.1016/j.resmic.2022.104025

5. Noble SM, Johnson AD. Strains and Strategies for Large-Scale Gene Deletion Studies of the Diploid Human Fungal Pathogen *Candida albicans*. Eukaryot Cell. 2005;4: 298–309. doi:10.1128/ec.4.2.298-309.2005
6. Walther A, Wendland J. PCR-based gene targeting in *Candida albicans*. Nat Protoc. 2008;3: nprot.2008.137. doi:10.1038/nprot.2008.137
7. Chauvel M, Nesseir A, Cabral V, Znaidi S, Goyard S, Bachellier-Bassi S, et al. A Versatile Overexpression Strategy in the Pathogenic Yeast *Candida albicans*: Identification of Regulators of Morphogenesis and Fitness. PLoS One. 2012;7: e45912. doi:10.1371/journal.pone.0045912
8. Homann OR, Dea J, Noble SM, Johnson AD. A Phenotypic Profile of the *Candida albicans* Regulatory Network. PLoS Genet. 2009;5: e1000783. doi:10.1371/journal.pgen.1000783
