# Supplementary material for: Metabolic reprogramming during Candida albicans planktonic-biofilm transition is modulated by the transcription factors Zcf15 and Zcf26
Source: PLoS Biol. 2024 Jun 21;22(6):e3002693. doi: 10.1371/journal.pbio.3002693 (PMC11221756; doi:10.1371/journal.pbio.3002693)
Supplement: S1 Table — (PDF) [file pbio.3002693.s010.pdf]

S1 Table. *C. albicans* strains and *E. coli* plasmids used in this study

Yeast strain	Genotype	Reference
SN76	*ura3Δ::λimm*^{434}*/ura3Δ::λimm*^{434} *iro1Δ::λimm*^{434}*/iro1Δ::λimm*^{434} *arg4Δ/arg4Δ his1Δ/his1Δ*	[1]
CEC4642	SN76 *ADH1/adh1::*P$_{TDH3}$*-carTA::SAT1 arg4Δ/CaARG4 his1Δ::hisG/HIS1*	This study
CEC4665	CEC4642 *RPS1/RPS1::*CIp10	This study
CEC6038	CEC4642 *RPS1/RPS1::*CIp10-P$_{TET}$*-ORF19.7199*	This study
CEC6039	CEC4642 *RPS1/RPS1::*CIp10-P$_{TET}$*-NRG1*	This study
CEC6040	CEC4642 *RPS1/RPS1::*CIp10-P$_{TET}$*CBF1*	This study
CEC6041	CEC4642 *RPS1/RPS1::*CIp10-P$_{TET}$*-GAL7*	This study
CEC6042	CEC4642 *RPS1/RPS1::*CIp10-P$_{TET}$*-ORF19.5933*	This study
CEC6043	CEC4642 *RPS1/RPS1::*CIp10-P$_{TET}$*-RBF1*	This study
CEC6044	CEC4642 *RPS1/RPS1::*CIp10-P$_{TET}$*-ZFU2*	This study
CEC6045	CEC4642 *RPS1/RPS1::*CIp10-P$_{TET}$*-ORF19.1666*	This study
CEC6046	CEC4642 *RPS1/RPS1::*CIp10-P$_{TET}$*-PAB1*	This study
CEC6047	CEC4642 *RPS1/RPS1::*CIp10-P$_{TET}$*-ORF19.2973*	This study
CEC6048	CEC4642 *RPS1/RPS1::*CIp10-P$_{TET}$*-3720*	This study
CEC6049	CEC4642 *RPS1/RPS1::*CIp10-P$_{TET}$*-FCP1*	This study
CEC6050	CEC4642 *RPS1/RPS1::*CIp10-P$_{TET}$*-ORF19.5381*	This study
CEC6051	CEC4642 *RPS1/RPS1::*CIp10-P$_{TET}$*-ZCF26*	This study
CEC6052	CEC4642 *RPS1/RPS1::*CIp10-P$_{TET}$*-ZCF15*	This study
CEC6053	CEC4642 *RPS1/RPS1::*CIp10-P$_{TET}$*-ZCF8*	This study
CEC5915	CEC4642 *RPS1/RPS1::*CIp10-P$_{TDH3}$*-ZCF15*	This study
CEC5916	CEC4642 *RPS1/RPS1::*CIp10-P$_{TDH3}$*-ZCF15*	This study
CEC5917	CEC4642 *RPS1/RPS1::*CIp10-P$_{TDH3}$*-ZCF26*	This study
CEC5918	CEC4642 *RPS1/RPS1::*CIp10-P$_{TDH3}$*-ZCF26*	This study
CEC5929	CEC4642 *RPS1/RPS1::*Clp10-P$_{TET}$*-TAP-ZCF15*	This study
CEC5930	CEC4642 *RPS1/RPS1::*Clp10-P$_{TET}$*-TAP-ZCF15*	This study
CEC5931	CEC4642 *RPS1/RPS1::*Clp10-P$_{TET}$*-TAP-ZCF26*	This study

CEC5932	CEC4642 *RPS1/RPS1::*Clp10-P_{TET}-*TAP-ZCF26*	This study
CEC6028	CEC4642 *RPS1/RPS1::*CIp10-P_{TET}-*ZCF25*	This study
SN152 *HIS1*/*LEU2*	*arg4Δ/arg4Δ leu2Δ/LEU2 his1Δ/HIS1 URA3/ura3Δ IRO1/iro1Δ*	[2]
TF099	*arg4Δ/arg4Δ leu2Δ/leu2Δ his1Δ/his1Δ URA3/ura3Δ IRO1/iro1Δ zcf15Δ::CmLEU2/zcf15::CdHIS1*	[2]
TF135	*arg4Δ/arg4Δ leu2Δ/leu2Δ his1Δ/his1Δ URA3/ura3Δ IRO1/iro1Δ zcf26Δ::CmLEU2/zcf26::CdHIS1*	[2]
CEC5935	SN152 *zcf26Δ::HIS1/zcf26Δ::LEU2 zcf15Δ::FRT/zcf15::FRT*	This study
**Plasmid**	**Description**	**References**
ECC1979	pSFS2A-5-UTR-3UTR-ZCF15	This study
ECC1983	CIp-pTET-NTAP-ZCF15	This study
ECC1984	CIp-pTET-NTAP-ZCF26	This study
ECC1985	CIp-pTDH3-ZCF15	This study
ECC1986	CIp-pTDH3-ZCF26	This study
ECC843	CIp-CaTDH3p-GTW	This study
ECC1095	CIp-pTET-rTAP-GTW-SP	[3]

References:

1. Noble SM, Johnson AD. Strains and Strategies for Large-Scale Gene Deletion Studies of the Diploid Human Fungal Pathogen *Candida albicans*. Eukaryot Cell. 2005;4: 298–309. doi:10.1128/ec.4.2.298-309.2005

2. Homann OR, Dea J, Noble SM, Johnson AD. A Phenotypic Profile of the *Candida albicans* Regulatory Network. PLoS Genet. 2009;5: e1000783. doi:10.1371/journal.pgen.1000783

3. Legrand M, Bachellier-Bassi S, Lee KK, Chaudhari Y, Tournu H, Arbogast L, et al. Generating genomic platforms to study *Candida albicans* pathogenesis. Nucleic Acids Res. 2018;46: 6935–6949. doi:10.1093/nar/gky594
