# Supplementary material for: Metabolic reprogramming during Candida albicans planktonic-biofilm transition is modulated by the transcription factors Zcf15 and Zcf26
Source: PLoS Biol. 2024 Jun 21;22(6):e3002693. doi: 10.1371/journal.pbio.3002693 (PMC11221756; doi:10.1371/journal.pbio.3002693)
Supplement: S2 Table — (PDF) [file pbio.3002693.s011.pdf]

S2 Table Oligonucleotide primers used in this study

Primer name	Sequence (5'-3')
TEC1qFP	GCAATTTCTGGGCAGATTG
TEC1qRP	TGGGAAATGTGCATTTAGG
Tye7qFP	GAATGACCTGGAAAAACCTAG
Tye7qRP	CAAATCTACTCTGAGAACAATC
IDP2qFP	CGTACACGGACACACACGAAAC
IDP2qRP	CATCGTCATTTTTTTCGCGTG
OSM2qFP	GGAGGGGGACACCGGAGAAAG
OSM2qRP	CGTTCCCGCGTCAAATTATCC
MDH1-3qFP	CGCAAAATGCTTGTTACACC
MDH1-3qRP	CGCCACCTTCGCAAACAAAC
orf19.4690-F	GAATATGCCGTTGGGTGTTT
orf19.4690-R	AACCCGAACCCAAAAACAAT
ZCF26FqRT	CATTGTTGTCCTTCCCGTTAC
ZCF26RqRT	GTCTTTTCTTTCATCGTCTGGTG
TEF3-F	GATCACAATTGGGTCCAAGG
TEF3-R	AGCAGCGGCAATCTTGTTAC
ZCF15FqRT	GAAAGGAGCGAGGAAGGAGAG
ZCFR15qRT	GTGGCTCTCGTTTCCCTGAATG
HWP2FqRT	CCCAGCATCTTCAACTACTAG
HWP2RqRT	CAGTGACAACAATAGCACC
HWP1RTFP	CACAACAGCCACAAGAACC
HWP1RTRP	AGGTTGAGGTGGATTGTCG
ECE1RTFP	CACCTACTGTTCCTGCACC
ECE1RTRP	ATTACTTGTGGAATGTTGCC
IHD1RTFP	GAATTGGCTCTGTGTGATTG
IHD1RTRP	ACTTGTAGATTGACCTTGAG
INO1RTFP	GCTGATGTTTTGCCAAATGTC
INO1RTRP	TTCCAAGATAGAAGCAACGGC

4571RTFP	TCAGTAAAGTTCCCCCATTTG
4571RTRP	ACAGAGTTCGAGCACTTTGAC
ZCF15USSATF	GGGGTACCGGTCCCTCGGATCAACAAG
ZCF15USSATR	CCGCTCGAGTTGAAATATTTGTGAATAG
ZCF15DSSATF	TTCCCGCGGTATAGACTCTTTTTTTACATATA
ZCF15DSSATR	CGAGCTCCCACCAATACTATTACTAC
CIpUL	ATACTACTGAAATTTCCTGACTTTC
CIpUR	ATTACTATTTACAATCAAAGGTGGTC
